# Revisiting Gaussian Markov random fields and Bayesian disease mapping

**DOI:** 10.1177/09622802221129040

**Published:** 2022-11-01

**Authors:** Ying C MacNab

**Affiliations:** School of Population and Public Health, 8166 University of British Columbia, Vancouver, Canada

**Keywords:** Bayesian disease mapping, Besag, York and Mollie (BYM) model, BYM (adaptive) reparameterization, conditional autoregressive models, deviance information criterion, Gaussian Markov random fields, local influence, scaling, spatial smoothing, spatial dependence, widely applicable information criterion

## Abstract

We revisit several conditionally formulated Gaussian Markov random fields, known as the intrinsic conditional autoregressive model, the proper conditional autoregressive model, and the Leroux et al. conditional autoregressive model, as well as convolution models such as the well known Besag, York and Mollie model, its (adaptive) re-parameterization, and its scaled alternatives, for their roles of modelling underlying spatial risks in Bayesian disease mapping. Analytic and simulation studies, with graphic visualizations, and disease mapping case studies, present insights and critique on these models for their nature and capacities in characterizing spatial dependencies, local influences, and spatial covariance and correlation functions, and in facilitating stabilized and efficient posterior risk prediction and inference. It is illustrated that these models are Gaussian (Markov) random fields of different spatial dependence, local influence, and (covariance) correlation functions and can play different and complementary roles in Bayesian disease mapping applications.

## 1 Introduction

In the Bayesian disease mapping literature, several conditionally formulated Gaussian Markov random fields (GMRF), also known as conditional autoregressive (CAR) models, have been proposed as spatial prior options for random effects in spatial generalized linear mixed effects (GLMM) models for spatially aggregated areal data.^1^ For mapping disease risks in small geographic areas, Bayesian GLMM Poisson models are typically used, in which the random effects represent log relative risks for geographic areas under study, e.g., counties or local health areas of a province or state or country; see Lawson^2^ and Martinez-Beneito and Botella-Rocamora.^3^ The present paper concerns with mapping of a single disease, for which the main goals are typically to ascertain geographical risk distribution of the disease and identify geographic areas of elevated (and lowered) disease risks. To achieve these goals, hierarchically formulated Bayesian GLMM is commonly used to model disease incidence or mortality data and to facilitate stabilized and efficient posterior risk prediction and inference.

Without essential loss of generality, we consider a typical disease mapping model, a hierarchically formulated Bayesian GLMM of Poisson likelihood for areal data of observed disease incidence or mortality cases, denoted $y=(y_{1},\ldots ,y_{n})$ for n contiguous geographic areas under study:

(1) $$y_{i}|E_{i},\gamma_{i}∼\text{Poisson}(\lambda_{i}),\text{log}(\lambda_{i})=\text{log}(E_{i})+\text{log}(\gamma_{i}),i=1,2,\ldots ,n,$$

(2) $$\text{log}(\gamma_{i})=\mu +\psi_{i},\text{E}(\psi_{i})=0,\forall i,$$

(3) ψ∼Pr(ψ|θ),

(4) μ∼Pr(μ),θ∼Pr(θ),

where $E=(E_{1},\ldots ,E_{n})$ , $E_{i}$ is the expected number of cases for area i ( i=1,2,…,n ), calculated using reference rate(s) and area-specific population at risk (usually subject to age and/or gender standardization), $\lambda =(\lambda_{1},\lambda_{2},\ldots ,\lambda_{n})$ is the n -vector of underlying relative risks, $\text{log}(E_{i})$ is the GLMM offset, μ is the GLMM intercept, $\psi =(\psi_{1},\ldots ,\psi_{n})$ is the n -vector of random variates (of zero mean). The main analytic efforts of Bayesian disease mapping typically involve (i) risk model specification for (3), where θ denotes risk model parameter(s), (ii) specification of (4), the priors for μ and θ , (iii) posterior estimation, learning, and inference of all unknowns, typically implemented via Markov Chain Monte Carlo simulations, and (iv) model evaluation. This paper concerns mainly the spatial risk model options for (3), which is one of the most important analytic considerations in disease mapping applications. We focus on three CAR models that are commonly used in Bayesian disease mapping, they are the well known intrinsic CAR (iCAR, Besag et al.^1^), proper CAR (pCAR, Cressie^4^), and Leroux et al. CAR (LCAR, Leroux et al.^5^). We also consider several convolution models, such as the Besag, Yorke, and Mollie (BYM) model^1^ and its re-parameterization named MBYM in MacNab,^6^ and their scaled alternatives.^7,8^

In MacNab,^6^ the three CARs, and the BYM and MBYM, were studied together as prior options in Bayesian disease mapping. The present study revisits and extends the topic by researching these models analytically, via graphic visualization, and with comprehensive simulation and case studies. The main objective, contribution, and message of the present paper is to shed light on the risk models discussed herein, not just as competing prior options for spatial smoothing but also as complementary risk models in disease mapping, spatial regression, and related studies. We do so by presenting important insights and critique on these models for their nature and capacities in modelling spatial dependencies, local influences, spatial correlations, and spatially structured heterogeneities, and in facilitating stabilized and efficient posterior risks prediction and inference based on data of extremely rare or rare or more common disease.

Another contribution of the present study is the illustration of a recent proposal of deviance information criterion (DIC^12,9–11^) and the widely applicable information criterion (WAIC^13–15^) for model evaluation and comparison via simulation and case studies. In addition, a new proposal of adaptive MBYM is put forward and illustrated in Bayesian disease mapping and spatial regression.

The rest of the paper is organized as follows. Section 2 reviews the above-mentioned risk models analytically and via graphical illustrations. Section 3 presents a comprehensive simulation study that further elucidates the risk models and associated Bayesian method for posterior estimation and inference. Section 4 presents the three case studies illustrating Bayesian disease mapping based on data of extremely small, small, modest, and large sample size, respectively. Section 5 concludes with a summary discussion.

## 2 Conditionally formulated Gaussian Markov random fields

### 2.1 A CAR construction and its three options of parameterization

The following Gaussian conditional mean and variance functions define a general construction of CARs:

(5) $$\text{E}(\psi_{i}|\psi_{-i})=\sum_{k∼i}B_{ik}\psi_{k},\text{Var}(\psi_{i}|\psi_{-i})=\sigma_{i}^{2},\text{\textbackslash{},for}\;i=1,2,\ldots ,n,$$

which, under consistency conditions,^16,17^ give rise to a unique GMRF Pr(ψ|θ) with precision and covariance matrices

(6) $$\Omega_{\psi }=\sigma^{-2}(I_{n}-B),\Sigma_{\psi }=(I_{n}-B{)}^{-1}\sigma^{2},$$

where $\sigma^{2}=\text{diag}(\sigma_{1}^{2},\sigma_{2}^{2},\ldots ,\sigma_{n}^{2})$ , $B=(B_{ik})$ is a n by n matrix that characterizes spatial dependencies; k∼i stands for the area i and k are neighbours and the $\psi_{i}$ and $\psi_{k}$ are conditionally dependent, given the rest of the $\psi_{j}$ s (for j≠i,k) ; $\psi_{-i}=(\psi_{1},\ldots ,\psi_{i-1},\psi_{i+1},\ldots \psi_{n})$ . In disease mapping, CARs are commonly defined on an irregular lattice of areal map, for which ‘neighbourhood’ is commonly defined by area-adjacency, e.g., areas that share common border(s) are neighbours.

GMRFs are *undirected* graphical models that characterize probabilistic interactions of *directly related variables*.^6,17^ Of the GMRFs commonly used in disease mapping (see Table 1), the B in expression (6) is typically a sparse matrix, with elements $B_{ik}\neq 0$ *if and only if* k∼i , where $B_{ik}$ quantifies conditional dependency and *direct influence* of area k on area i , provided the two areas are neighbours. We name hereafter B the *spatial dependence matrix* and the coefficients $\left\{B_{ik},\forall k∼i\right\}$ of $\text{E}(\psi_{i}|\psi_{-i})$ in (5) the *coefficients of influence*.^18^

**Table 1.** Options of model parameterization. W is the well-known ‘neighbourhood’ connectivity matrix: $W=(w_{ik}),w_{ik}=1$ when i∼k or $w_{ik}=0$ otherwise; $w_{i+}=\sum_{k∼i}w_{ik},D_{w}=\text{diag}(w_{1+},\ldots w_{n+})$ , $w_{i+}^{c}=1-c+cw_{i+}$ , $Q(c)=(D_{w}-cW)$ . ${}^{1}$ : $\psi^{s}∼\text{iCAR}(\sigma_{s}^{2}),\psi^{h}∼\text{IIDN}(\sigma_{h}^{2})$ ; ${}^{2}$ : $\phi^{s}∼\text{iCAR}(\sigma^{2}),\phi^{h}∼\text{IIDN}(\sigma^{2})$ , IIDN: independent and identically distributed normal distribution; ${}^{3}$ : Corpas-Burgos and Martinez-Beneito,^19^ $\sigma_{s}=(\sigma c_{1}^{1/2},\sigma c_{2}^{1/2},\ldots ,\sigma c_{n}^{1/2})$ ; ${}^{4}$ : $c=\text{diag}(c_{1},c_{2},\ldots ,c_{n})$ .

Model	$B_{ik}$	Var $\left(\psi_{i}|\psi_{-i}\right)$	$\Omega_{\psi }$	The role of c or c
pCAR( $c,\sigma^{2}$ )	$\frac{c}{w_{i+}}$	$\frac{\sigma^{2}}{w_{i+}}$	$\sigma^{-2}Q(c)$	Smoothing
Cressie^4^				
LCAR( $c,\sigma^{2}$ )	$\frac{c}{w_{i+}^{c}}$	$\frac{\sigma^{2}}{w_{i+}^{c}}$	$\sigma^{-2}(cQ(1)+(1-c)I_{n})$	Smoothing
Lerox et al.^5^				
iCAR( $\sigma^{2}$ )	$\frac{1}{w_{i+}}$	$\frac{\sigma^{2}}{w_{i+}}$	$\sigma^{-2}Q(1)$	
Besag et al.^1^				
	ψ re-parameterized		$\Sigma_{\psi }$	
BYM( $\sigma_{s},\sigma_{h}$ ) ${}^{1}$	$\psi =\psi^{s}+\psi^{h}$		$\sigma_{s}^{2}Q(1{)}^{-1}+\sigma_{h}^{2}I_{n}$	
Besag et al.^1^				
MBYM( c,σ ) ${}^{2}$	$\psi =\sqrt{c}\phi^{s}+\sqrt{1-c}\phi^{h}$		$\sigma^{2}(cQ(1{)}^{-1}+(1-c)I_{n})$	Smoothing
MacNab^6^				
BYM( $\sigma_{s},\sigma_{h}$ ) ${}^{3}$	$\psi =\psi^{s}+\psi^{h}$		$\sigma_{s}Q(1{)}^{-1}\sigma_{s}^{⊤}+\sigma_{h}^{2}I_{n}$	Heterogeneities/discontinuities
(C-B 2020)				
MBYM( c,σ ) ${}^{4}$	$\psi_{i}=\sqrt{c_{i}}\phi_{i}^{s}+\sqrt{1-c_{i}}\phi_{i}^{h}$		$\sigma^{2}(c^{1/2}Q(1{)}^{-1}c^{1/2}+(I_{n}-c))$	Heterogeneities/discontinuities
(new proposal)				

pCAR: proper conditional autoregressive model; LCAR: Leroux et al. conditional autoregressive model; iCAR: intrinsic conditional autoregressive model; BYM: Besag, Yorke, and Mollie; MBYM: Modified Besag, Yorke, and Mollie.

The GMRF precision matrix (6) must be symmetric and non-negative definite. To fulfil the two requirements, functional characterizations and simplified parameterizations to the CAR conditionals have been proposed (mainly) in the disease mapping literature; and the previously mentioned iCAR, pCAR, and LCAR are the most commonly used GMRFs in disease mapping applications at the present time; see Table 1 for the CAR specifications, where key references are given. The three CAR formulations and parameterizations are commonly viewed as competing spatial risk priors; each has its strength and limitations, which we discuss and illustrate in this section.

The CARs were initially proposed to facilitate borrowing information and spatial smoothing.^1,4,5^ However, shown in MacNab,^18^ and in the present paper, as we broaden the scope of Bayesian disease mapping to the studies of rare or more common diseases, and non-communicable or communicable diseases, CARs and GMRFs can offer tools not just for borrowing information and spatial smoothing, but for analysis of spatial risk dependencies, local risk influences, spatial risk correlations, and spatial risk heterogeneities.

### 2.2 The pCAR and LCAR: Spatial dependence and local influence

**Figure 1.**
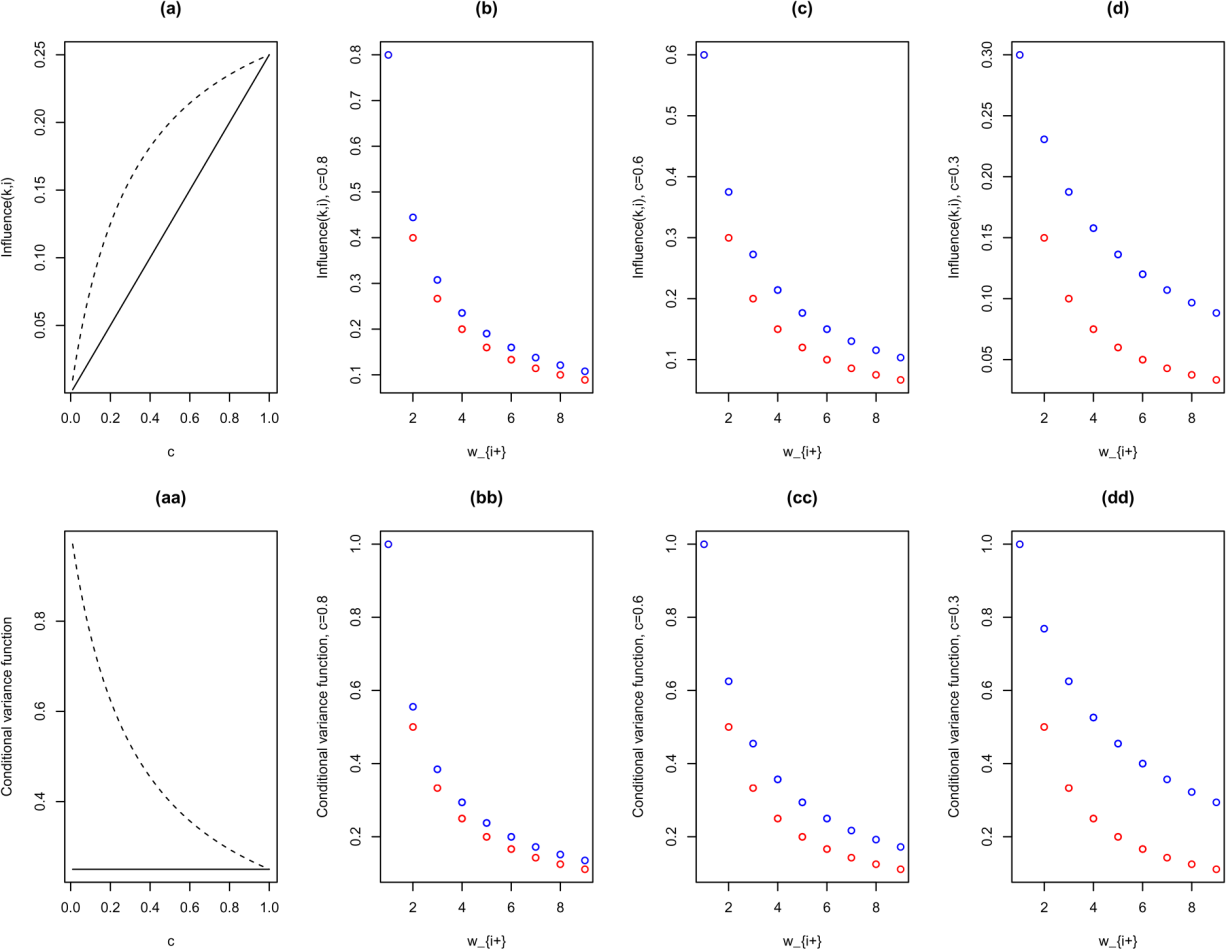
pCAR (c,σ) and LCAR (c,σ) influence functions and conditional variance functions illustrated, for σ=1 and $w_{i+}=4$ (for plots (a) and (aa)). The solid line: pCAR, the dashed line: LCAR. Red dot: pCAR, Blue dot: LCAR. pCAR: proper conditional autoregressive model and LCAR: Leroux et al. conditional autoregressive model.

The pCAR( c,σ ) and LCAR( c,σ ) conditionals lead to full rank GMRFs, where c and σ are the respective spatial dependence and scale parameters. The coefficients of influence in the pCAR and LCAR conditional means are functions of c , where $B_{ik}=B_{ik}(c),\forall k∼i$ , is simply named the *influence functions*, denoted Influence (k,i) hereafter.^18^ The two CARs share a common characteristic that they postulate asymmetric conditional dependency (i.e. asymmetric direct influence) of $\psi_{k}$ on $\psi_{i}$ versus $\psi_{i}$ on $\psi_{k}$ , provided $w_{i+}\neq w_{k+}$ , i∼k , and 0<c<1 , where $w_{i+}$ , defined in Table 1, is the number of neighbours of area i . Further, their influence functions imply that the *direct influence* of the area k on its neighbouring area i is inversely proportional to the neighbourhood size of *the* area i (also see Figure 1): An area with a higher number of neighbours is less influenced by its neighbour who has a lower number of neighbours. As noted in MacNab,^18^ this could be an intuitively plausible assumption, consistent with their conditional precision functions (see Table 1): One might expect that an area of higher precision of risk prediction should be less influenced by an area with a lower precision of (predicted) risk. The spatial dependence parameter c in pCAR or LCAR is often called a *spatial smoothing parameter*; it regulates local (i.e. within neighbourhood) risks smoothing over the map. The two CARs also have iCAR( σ ) as their limiting distribution when the spatial parameter c tends to 1.

As Figure 1 illustrates, the pCAR influence function, Influence $(k,i)=c/w_{i+},\forall k∼i$ , is a linear function of c , whereas the LCAR influence function, Influence $(k,i)=c/(1-c+c\;w_{i+}),\forall k∼i$ , is a non-linear function of c . The pCAR and LCAR influence functions are comparable for large c (e.g. c≥0.8 ).

In Bayesian disease mapping, the spatial parameter c in pCAR or LCAR is often contained in (0,1) to hypothesize positive spatial dependencies and correlations and for borrowing information and spatial smoothing. However, the pCAR( c,σ ) is a valid GMRF when c in contained in $(c_{\text{min}},c_{\text{max}}$ ), where $c_{\text{min}}<0$ and $c_{\text{max}}=1$ are the reciprocals of the minimum and maximum eigenvalues of $D_{w}^{-1/2}WD_{w}^{-1/2}$ .^20^

In LCAR, the spatial dependence parameter c , with c∈(0,1) , is also known as a spatial *weight* parameter: It weights a precision matrix of the iCAR( σ ) and a precision matrix of n independent and identically distributed normal (IIDN) variates, denoted IIDN( $\sigma^{2}I_{n}$ ),^5^ where $I_{n}$ is the identity matrix of n -dimension. LCAR is also interpreted as a mixing of purely local (spatial) and global (non-spatial) smoothing.^21^

The LCAR is often favoured over the pCAR for the fact that, when c=0 , the LCAR reduces to a independent and identical Gaussian prior with conditional variance $\sigma^{2}$ for all areas,^6,22^ whereas the pCAR reduces to n independent Gaussian priors with area-specific conditional variance $\sigma^{2}/w_{i+},\forall i$ .

The scale parameter σ and the spatial parameter c in LCAR together control the risk prediction variances and precisions (see Figure 1(aa)), as well as the resulting risks variability/heterogeneity over the map. The LCAR parameterization is also noted in MacNab^23^ as an ‘entangled’ spatial and non-spatial parameterization, and this ‘entanglement’ complicates and limits ones options for multivariate generalizations of LCAR. On the other hand, the pCAR spatial and scale parameters play separate and different roles: One regulates spatial dependencies, the other controls risk prediction variances and risks variability/heterogeneity over the map. As a consequence, the pCAR has rich options of multivariate and adaptive generalizations that have theoretical and practical appeals for modelling and interpreting multidimensional (cross) spatial dependencies and heterogeneities.^18,23^

### 2.3 The iCAR and convolution models: Spatially structured or clustered heterogeneity

The one-parameter iCAR( σ ) is typically considered as a pure spatial smoother; it is the pCAR and LCAR with c=1 . The iCAR rank n−1 precision matrix implies that the iCAR conditionals typically determine n risks under additional constraint, most commonly $\sum \psi_{i}=0$ . Another interpretation of the iCAR is via its Gaussian density^1^

(7) $$f(\psi |\sigma )\propto \text{exp}\left[-\sum_{k∼i}\frac{(\psi_{i}-\psi_{k}{)}^{2}}{\sigma^{2}}\right],$$

which models spatially structured risk variation over the map via pair-wise risks differences of neighbouring areas, regulated by the scale parameter σ in (7). For this reason, the iCAR( σ ) is commonly motivated as a spatial risk prior for modelling spatially *structured or clustered heterogeneity*.^1^

In disease mapping, the iCAR (7) is commonly used when the n -vector of random effects ψ is modelled as $\psi =\psi^{s}+\psi^{h}$ , where $\psi^{s}∼\text{iCAR}(\sigma_{s}^{2})$ for modelling spatially structured (clustered) heterogeneity or effects of omitted covariates that are *spatially varying*, and $\psi^{h}∼\text{IIDN}(\sigma_{h}^{2})$ , for modelling extra-Poisson variation or effects of omitted covariates that are *randomly varying*. This is a convolution model, well known as the Besag, Yorke, and Mollie (BYM) model.^1^ The BYM model is also noted for its excessive parameterization and identification issues.^6,24^

In the present paper, we illustrate that, to gain identification, posterior estimation and inference of BYM can be implemented by placing (weakly) informative priors on the BYM scale parameters or by a reparameterization of BYM, named modified BYM or MBYM in MacNab^6^:

(8) $$\psi^{s}=\sqrt{c}\phi^{s},\psi^{h}=\sqrt{1-c}\phi^{h},$$

where $\phi^{s}∼\text{iCAR}(\sigma^{2})$ , $\phi^{h}∼\text{IIDN}(\sigma^{2}I_{n})$ , c∈(0,1) is a weight parameter such that the covariance matrix of ψ is a weighted sum of the covariance matrices of $\phi^{s}$ and $\phi^{h}$ ; also see Table 1 for the BYM and MBYM covariance matrices, respectively.

While not discussed in the literature, in the present paper we also highlight and critique on the re-parameterization approach to identification by noting that it is equivalent to placing functional constraints on the BYM scale parameters $\sigma_{s}=\sqrt{c}\;\sigma$ and $\sigma_{h}=\sqrt{1-c}\;\sigma$ , which also has its limitations and identification challenges. We return to the BYM or MBYM (abbreviated (M)BYM) identification issues again in Sections 3 and 4, where via simulation and case studies, Bayesian estimation and inference of BYM and MBYM in small, modest, and large sample settings are illustrated and evaluated.

A scaled iCAR( $\tau_{s}$ ), where $\tau_{s}=s^{2}\tau$ is the scaled precision, was proposed in Sorbye and Rue^25^ for mapping the iCAR precision $\tau_{s}$ to marginal standard deviation of the iCAR covariance matrix $\Sigma^{\text{scaled}}=(\tau_{s}(D_{w}-W){)}^{-1}$ , where $\Sigma^{\text{scaled}}$ is the generalized inverse of the iCAR precision matrix, $s=\text{exp}(\frac{1}{2n}\sum_{i=1}^{n}\text{log}(\Sigma^{*}[i,i]))$ is named a ‘reference standard deviation’, $\Sigma^{*}$ is the generalized inverse of $D_{w}-W$ . The scaled iCAR was motivated for the interpretation of sσ ( $\sigma =1/\sqrt{\tau }$ ) as approximating the marginal standard deviation of all components of ψ and for Bayesian estimation and inference of τ under informative Gamma hyper-prior. Simpson et al.^8^ put forward a proposal of scaled BYM, also named BYM2 in Riebler et al.,^7^ which is an equivalent of the scaled MBYM:

(9) $$\psi =\sqrt{c}\;{\tilde{\psi }}^{s}+\sqrt{1-c}\;{\tilde{\psi }}^{h},$$

where ${\tilde{\psi }}^{s}∼\text{scaled iCAR}(\tau_{s})$ and ${\tilde{\psi }}^{h}∼\text{IIDN}(\tau )$ .

### 2.4 The CAR and (M)BYM covariance matrices

The CAR conditionals (5), and the resulting sparse precision matrix Ω in (6), characterize local properties of a GMRF. They quantify direct relationships between an area and its neighbours, such as spatial risk dependencies, local risk influences, partial correlation, and risk prediction precisions and variances. The resulting GMRF covariance matrix defines the GMRF (marginal) correlation, variance, and covariance functions. It is well known that the GMRF covariance matrix (6), commonly derived as the (general) inverse of the precision matrix for conditionally specified GMRF, does not have analytically transparent expressions for its dense elements,^6,17,26,27^ that is, analytically illustrative interpretations of GMRF (correlation) covariance functions are often not available.

**Figure 2.**
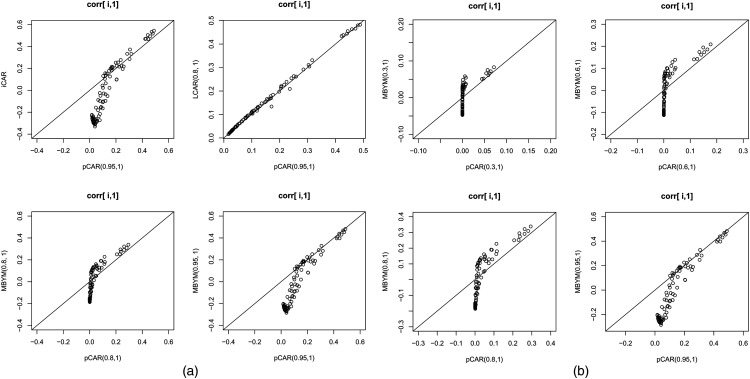
Illustrative spatial correlation functions for the pCAR, LCAR, iCAR and MBYM, respectively, with indicated parameter values. The spatial correlation functions display correlations between county 1 and county i , for all i≠1 . The Minnesota county map. pCAR: proper conditional autoregressive model; LCAR: Leroux et al. conditional autoregressive model; iCAR: intrinsic conditional autoregressive model; MBYM: modified Besag, Yorke, and Mollie.

However, some of the characteristics of the GMRF covariance matrices, as well as the (M)BYM covariance matrices, can be explored and understood by graphic visualization, such as plots of spatial correlation and covariance functions, as well as the marginal variance functions, for given model parameters. Here, we define spatial correlation or covariance functions as correlation $\left(\psi_{i},\psi_{k}\right)$ , or covariance $\left(\psi_{i},\psi_{k}\right)$ , for given k and all i≠k . That is, we define spatial correlation (covariance) functions as marginal correlation (covariance) functions with respect to area k , ∀k , and its m th-order neighbours, for $m=1,2,\ldots ,M_{k}$ ,^6^ as illustrated in Figure 2 and Figures S1 and S2 in the Supplemental Material (SM) to the paper using the county-level map of Minnesota (USA).^28,29^ As we illustrate herein, graphic visualization can shed light on the GMRF spatial correlation or covariance functions and unveil different spatial features and patterns of spatial correlation (covariance) functions for the risk models as Gaussian Markov random fields for iCAR, pCAR and LCAR or Gaussian random field for (M)BYM.

Specifically, for iCAR, pCAR, LCAR and (M)BYM, Figure 2, and the supplement Figures S1 and S2 display spatial correlation functions between county 1 and county i , for all i≠1 . Each of the correlation plots shows a cluster of notably higher correlations between the county and its first-order neighbours (county 1 has eight first-order neighbours), with *decreasing* correlations between county 1 and its m th-order neighbours for *increasing* m . The correlation and variance plots also indicate that for large c the four models are comparable spatial smoothers; c controls the smoothness of the risk map. Of note, for small or large c , c∈(0,1) , the pCAR and MBYM assume comparable within neighbourhood spatial correlations (see Figure 2).

Illustrated in the supplement Figure S1, even for small values of spatial parameters, the pCAR and LCAR model locally clustered spatial correlation functions. It also shows that the differences between pCAR and LCAR correlation functions are consistent with the differences in their influence functions (see Figure 1). For c∈(0,1) , the LCAR allows for higher spatial (influences) dependencies and correlations to be modelled with the same value of c .

In addition, the supplement Figures S1 and S2 illustrate that the iCAR and (M)BYM (of first-order adjacency-defined neighbourhood map) lead to positive and clustered spatial (correlation) covariances between an area and its first-order neighbours but negative (correlations) covariances between an area and its ‘distance’ m th-order neighbours, ‘distance’ in terms of high order m (i.e. areas that are further apart). The supplement Figure S1 shows that the iCAR and the pCAR (LCAR) of large spatial parameter (e.g. c=0.95 for pCAR and 0.8 for LCAR) postulate comparable clustered positive correlations between first-order neighbours.

Further more, the supplement Figure S2 offers new insight into the (M)BYM partial corrrelation functions: They are spatially varying functions that lead to locally clustered partial correlations when the spatially structure variation exceed the unstructured variation (e.g. λ>0.6 ). Similar to the marginal correlation coefficents, the partial correlation coefficients are positive between an area and its first-order neighbours but negative between an area and its ‘distance’ m th-order neighbours.

### 2.5 A new proposal of adaptive convolution model

To allow for more flexible characterizations of spatial dependencies, local influences, and spatial heterogeneities/discontinuities, extensions of the CARs to adaptive spatial dependence parameterizations ( c,σ ) or adaptive spatial dependence and heterogeneity parameterizations ( c,σ ), as well as adaptive iCAR( σ ), have been proposed in the literature, where $c=(c_{1},c_{2},\ldots ,c_{n})$ and $\sigma =(\sigma_{1},\sigma_{2},\ldots ,\sigma_{n})$ ; see MacNab^18^ for a recent review.

In the context of Bayesian disease mapping, an adaptive BYM, defined by iCAR( $\sigma_{s}$ ) + IIDN( $\sigma_{h}$ ), was recently proposed in Corpas-Burgos and Martinez-Beneito,^19^ where $\sigma_{s}=(\sigma_{s}c_{1}^{1/2},\sigma_{s}c_{2}^{1/2},\ldots ,\sigma_{s}c_{n}^{1/2})$ , and the locally varying parameters c can introduce roughness to the spatial components (Table 1). A limitation of this adaptive BYM proposal is its identification issue, particularly for its use in univariate disease mapping, as mentioned in Corpas-Burgos and Martinez-Beneito^19^ and also in our experience of testing the model on several real-life univariate disease mapping data. In Corpas-Burgos and Martinez-Beneito,^19^ the adaptive BYM was implemented in mapping multivariate disease outcomes.

Here, we propose and illustrate (via a case study) an adaptive MBYM( c,σ ) parameterization in (8) and (9), respectively, where $c=(c_{1},c_{2},\ldots ,c_{n})$ (Table 1). In addition to gaining identifiability, another feature of the new adaptive MBYM is that both the $\psi^{s}$ and $\psi^{h}$ are modelled adaptively: $\psi^{s}∼\text{iCAR}(\sigma_{s})$ , $\psi^{h}∼\text{IDN}(\sigma_{h})$ , where $\sigma_{s}=\text{diag}(\sqrt{c_{1}}\;\sigma ,\sqrt{c_{2}}\;\sigma ,\ldots ,\sqrt{1-c_{n}}\;\sigma )$ , $\sigma_{h}=\mathrm{diag}(\sqrt{1-c_{1}}\sigma ,\sqrt{1-c_{2}}\sigma ,...,\sqrt{1-c_{n}}\sigma )$ . Notice that the locally varying c can introduce roughness to both the spatial and non-spatial components and the resulting risk map.

## 3 The simulation study

We carried out a comprehensive simulation study in the context of hierarchical Bayesian estimation, learning, and inference of GLMM (1)–(4), for options of the non-adaptive CAR or convolution risk models as random effects prior. Posteriors of all unknowns were estimated via Markov chain Monte Carlo (MCMC) simulations implemented in **WinBUGS**.^30^ It is worth mentioning that formulating GMRFs via full conditionals facilitates coding the powerful Gibbs sampler as a computational tool for posterior estimation of the spatial random effects with CAR/GMRF priors via MCMC simulations.^1,31^

The simulation and computational details are presented in the Supplemental Material (SM) to the paper. Simulated data were generated to represent disease mapping scenarios of extremely small, small, modest, or large sample sizes (as of expected disease counts, see SM for details). Detailed results are also presented in the SM, including seven tables (named the supplement Tables S1 to S7) and 14 figures (named the supplement Figures S3 to S16). Here, the key results are summarized and highlighted. The overall performances of posterior estimation and inference of model parameters and relative risks (and $\psi^{s}$ and $\psi^{h}$ ) are discussed in terms of posterior bias, root mean squared error (rmse), and coverage rate of the 95% credible interval.

Overall, the pCAR, LCAR and (scaled) MBYM led to comparable performances in terms of posterior estimation of the spatial dependence or weight parameter: Under non-informative prior c∼Beta(1,1) for c∈(0,1) , all indicating a tendency of underestimating a large spatial parameter or over-estimating a small spatial parameter, with considerable posterior uncertainties. Posterior biases and uncertainties decreased as the sample size increased or when informative priors for c were used. Small or modest posterior biases and comparable performances were observed from posterior estimation of the scale parameter σ , where modest posterior biases were observed for data of extremely small sample size.

For all simulation scenarios, the iCAR scale parameter was estimated with near-zero posterior bias and near-the-target coverage rate.

Consistent and comparable performances were observed from the posterior estimates of the (M)BYM and scaled (M)BYM model parameters. The (scaled) BYM was shown to perform slightly better than the (scaled) MBYM, observed from modestly lower posterior bias and rmse from the (scaled) BYM; this is particularly evident for data of extremely small sample size, likely as a result that the spatial weight parameter in MBYM is typically underestimated for a large c and over estimated for a small c . For data of modest or large sample size, the scaled and unscaled (M)BYM led to comparable posterior bias, rmse, and coverage rate for all model parameters.

Overall, the CAR and (M)BYM models led to consistent and comparable performances in terms of posterior risk prediction and inference; minor or modest differences were only observed for data of extremely small sample size. For all simulation scenarios, the iCAR performed well in terms of posterior risk prediction and inference. For all models and simulation scenarios, and even for extremely small sample size, the 95% credible intervals for the county-specific relative risks led to near or above 90% coverage rates.

For pCAR, LCAR and MBYM, minor or modest posterior risk sensitivities to spatial parameter prior options were mostly observed from the posterior risk standard deviations (posterior risk uncertainties) and the resulting posterior risk coverage rates; posterior risk biases and root mean square errors remained robust; informative spatial parameter priors led to reduced posterior risk standard deviations and improved posterior risk coverage rates.

In terms of posterior prediction and inference for relative risks (RRs) and the components $\psi^{s}$ and $\psi^{h}$ , comparable performances were also observed between BYM and MBYM, scaled BYM and scaled MBYM, and between the scaled and unscaled BYM or MBYM; modest differences were only observed from data of extremely small sample size (see SM for details). For (scaled) MBYM, informative spatial parameter priors led to reduced posterior standard deviations (posterior uncertainties) and improved posterior coverage rates for the relative risks and the respective components $\psi^{s}$ and $\psi^{h}$ .

For the five risk models, we present here illustrative simulation results of deviance information criterion, the Dbar (deviance), pD, and DIC scores, where pD = pD1 and pD1 is the *number of free parameters* defined in MacNab,^10^ in which the deviance, pD1 and DIC = Dbar+pD1 are invariant to re-parameterization and can facilitate model evaluation and comparison among (multivariate) CAR models, including those with non-identifiable or partially identifiable model parameter(s). Illustrative results of widely applicable information criterion (WAIC) were also presented, where WAIC = -2 lppd (predictive accuracy) + $2p_{\text{WAIC 2}}$ (effective number of parameters), lppd is the abbreviation of log point-wise predictive density; see Gelman et al.^13^ for details. Both the DIC and WAIC were calculated based on conditional likelihood of the Poisson data model.

The estimated DIC and WAIC statistics (e.g. mean scores and associated standard deviations) are overall comparable across the models, although the effective numbers of parameters in DIC (denoted pD) were consistently lower than those in WAIC (denoted pW); see Tables 2 and 3 and the supplement Figure S13 to S16 for illustrative results of simulation scenarios 1a and 2a. The two tables also present rates of true models preferred based on the estimated DIC and WAIC, respectively: The DIC-based rates may inform on model comparison in term of prediction accuracy of observed data (via deviance) and within-sample risk predictions, whereas the WAIC-based rates may inform on selection/comparison in term of out-of sample prediction accuracy (e.g. prediction accuracy of new counts and risks when new data is used).

**Table 2.** Selected DIC and WAIC results of the simulation study (Part I), c∼Beta(1,1) for all models. Rate ${}^{†}$ : Rate of true model prefered based on the estimated DIC and WAIC, respectively.

			pD		DIC		pW		WAIC		Rate ${}^{†}$	Rate ${}^{†}$
True model	Fitted model	Scenario	Mean	sd	Mean	sd	Mean	sd	Mean	sd	DIC	WAIC
pCAR	pCAR	S 1a	25	7	483	10	40	10	482	11		
pCAR	pCAR	S 2a	57	4	729	6	74	4	722	6		
pCAR	LCAR	S 1a	23	7	483	10	39	11	483	11	0.59	0.72
pCAR	LCAR	S 2a	56	4	729	6	75	5	723	7	0.44	0.77
pCAR	MBYM	S 1a	24	7	483	10	39	10	483	11	0.68	0.52
pCAR	MBYM	S 2a	56	4	729	6	74	5	722	7	0.69	0.46
pCAR	BYM	S 1a	25	7	483	10	41	10	483	11	0.64	0.53
pCAR	BYM	S 2a	56	4	729	6	75	5	723	6	0.66	0.72
pCAR	iCAR	S 1a	21	7	485	11	38	12	487	12	0.82	0.93
pCAR	iCAR	S 2a	55	4	732	7	79	6	730	8	0.99	1.00
LCAR	LCAR	S 1a	31	6	488	11	48	9	487	11		
LCAR	LCAR	S 2a	60	3	732	8	76	4	724	8		
LCAR	pCAR	S 1a	32	7	488	10	49	9	487	11	0.67	0.46
LCAR	pCAR	S 2a	61	3	732	8	76	4	723	8	0.66	0.28
LCAR	MBYM	S 1a	31	7	489	11	48	9	486	11	0.32	0.24
LCAR	MBYM	S 2a	60	3	732	8	75	4	722	8	0.12	0.12
LCAR	BYM	S 1a	33	6	489	10	50	8	488	10	0.65	0.46
LCAR	BYM	S 2a	60	3	732	8	77	4	724	8	0.77	0.61
LCAR	iCAR	S 1a	29	7	490	11	48	10	491	13	0.86	0.96
LCAR	iCAR	S 2a	59	4	735	8	81	5	731	9	1.00	1.00

pCAR: proper conditional autoregressive model; LCAR: Leroux et al. conditional autoregressive model; iCAR: intrinsic conditional autoregressive model; BYM: Besag, Yorke, and Mollie; MBYM: modified Besag, Yorke, and Mollie; DIC: deviance information criterion; WAIC: widely applicable information criterion.

**Table 3.** Selected DIC and WAIC results of the simulation study (Part II), c∼Beta(1,1) for all MBYM. Rate ${}^{†}$ : Rate of true model prefered based on the estimated DIC and WAIC, respectively.

			pD		DIC		pW		WAIC		Rate ${}^{†}$	Rate ${}^{†}$
True model	Fitted model	Scenario	Mean	sd	Mean	sd	Mean	sd	Mean	sd	DIC	WAIC
iCAR	iCAR	S 1a	28	6	487	10	46	9	488	11		
iCAR	iCAR	S 2a	58	4	732	6	78	5	726	7		
iCAR	MBYM	S 1a	40	5	498	8	45	8	483	10	0.12	0.03
iCAR	MBYM	S 2a	66	3	738	4	73	5	719	6	0.00	0.00
iCAR	BYM	S 1a	31	7	489	11	47	8	485	9	0.52	0.08
iCAR	BYM	S 2a	60	3	732	8	74	4	721	5	0.03	0.00
iCAR	pCAR	S 1a	32	6	487	8	47	8	485	9	0.65	0.28
iCAR	pCAR	S 2a	59	3	730	5	74	4	720	6	0.04	0.00
iCAR	LCAR	S 1a	40	5	498	8	46	8	485	10	0.45	0.13
iCAR	LCAR	S 2a	66	3	739	4	74	4	721	6	0.01	0.00
MBYM	MBYM	S 1a	40	5	497	8	59	7	495	9		
MBYM	MBYM	S 2a	65	3	737	4	80	3	726	5		
MBYM	BYM	S 1a	41	5	498	8	60	7	496	8	0.91	0.74
MBYM	BYM	S 2a	66	3	738	4	80	4	727	5	0.92	0.68
MBYM	iCAR	S 1a	38	6	501	9	62	8	503	11	0.99	1.00
MBYM	iCAR	S 2a	66	3	743	5	87	5	736	7	1.00	1.00
MBYM	pCAR	S 1a	41	5	499	8	60	7	496	8	0.79	0.74
MBYM	pCAR	S 2a	67	3	739	4	80	3	727	5	0.90	0.65
MBYM	LCAR	S 1a	40	5	498	8	60	7	497	9	0.85	0.87
MBYM	LCAR	S 2a	66	3	739	4	81	4	727	5	0.90	0.72

pCAR: proper conditional autoregressive model; LCAR: Leroux et al. conditional autoregressive model; iCAR: intrinsic conditional autoregressive model; BYM: Besag, Yorke, and Mollie; MBYM: modified Besag, Yorke, and Mollie; DIC: deviance information criterion; WAIC: widely applicable information criterion.

Overall, when pCAR or LCAR or (M)BYM was the true risk model, the estimated DIC and WAIC scores led to consistent and high rates of favouring correct models when iCAR was the misspecified prior. When iCAR is the true risk models, the WAIC scores led to high rates of favouring pCAR, LCAR, and (M)BYM for both scenarios; this may suggest evidence that the iCAR is not a preferred out-of-sample predictive model among the five.

When the (M)BYM was the true data generating risk model, the DIC- and WAIC rates of true model preferred were consistent and comparably high (e.g. favouring the correct model), which suggested evidence that the (M)BYM may be a plausible risk model for both the within- and out-of-sample predictions.

## 4 The three case studies

The three case studies are presented herein to illustrate, via results of Bayesian GLMM (1)–(4), Bayesian risk mapping of extremely rare, rare, and more common diseases, represented by areal data of small, modest, or large (expected) counts (see Table 4 for summary statistics). The first case study was a re-analysis of the Jin et al.^28^ cancer mortality data for 87 counties of Minnesota (USA). This data set contains county-level death counts (observed and expected) for cancer of oesophagus ( $y_{1}$ ) and lung ( $y_{2}$ ), respectively; they are illustrative examples of data of a rare (esophagus) and a more common (lung) cancer. The second is an analysis of the West Yorkshire (UK) ward-level counts of incidence cases for cancer of the oral cavity ( $y_{1}$ ) and lung ( $y_{2}$ ), respectively. The data set is made available in the GeoBUGS^31^; oral cavity cancer is an example of rare disease. The third example is a re-analysis of the COVID-19 infection data for the counties of Minnesota (USA), previously analyzed in MacNab.^18^ The analysis presented herein serves as an illustrative example of disease risk mapping based on data of comparably large counts of infection cases, without or with covariates. The case study III also illustrates applications of the adaptive MBYM and its scaled alternative in modelling COVID-19 infection risks without or with covariates.

**Table 4.** Data for the three illustrative case studies: Summary statistics of areal-level counts.

		Observed counts	Expected counts
Case study	Variable	(min, median, max)	(min, median, max)
I	$y_{1}$	(0, 12, 439)	(2.4, 11.2, 418.5)
I	$y_{2}$	(24, 117, 5294)	(28.7, 131.7, 4901.3)
II	$y_{1}$	(1, 6, 14)	(3.2, 5.6, 9.9)
II	$y_{2}$	(68, 115, 300)	(63.5, 115.9, 217)
III	y	(118, 2041, 98278)	(296.3, 1840.6, 105420.8)

### 4.1 Disease risk mapping without covariates

Posterior estimates (median and standard deviation) of the model parameters for all case studies are presented in the supplement Table S8, where modest to high spatial risk dependencies were suggested from the CAR models (indicated by modest to high posterior estimates of the spatial parameters). Consistent with the results of the simulation study, modest (although noteworthy) posterior sensitivities to spatial parameter prior specifications were observed from the first two case studies; Table 5 illustrates the results of pCAR. Modest posterior risk sensitivity was only observed from the case study II; Supplemental Figure S17 illustrates the results of pCAR.

**Table 5.** Posterior estimates, median and standard deviation (sd), of model parameters under the indicated informative or non-informative hyperprior for c in pCAR( c,σ ).

	Case Study I				Case Study II			
	c∼Beta(1,1)		c∼Beta(8,3)		c∼Beta(1,1)		c∼Beta(8,3)	
Parameter	Median	sd	Median	sd	Median	sd	Median	sd
$c_{1}$	0.75	0.25	0.77	0.12	0.65	0.28	0.76	0.13
$c_{2}$	0.94	0.06	0.89	0.06	0.93	0.07	0.87	0.07
$\sigma_{1}$	0.32	0.10	0.33	0.10	0.30	0.14	0.30	0.14
$\sigma_{2}$	0.20	0.03	0.22	0.03	0.39	0.03	0.40	0.03
$\mu_{1}$	− 0.05	0.05	− 0.05	0.05	0.00	0.04	− 0.01	0.04
$\mu_{2}$	− 0.07	0.04	− 0.08	0.03	− 0.02	0.05	− 0.02	0.04

pCAR: proper conditional autoregressive model.

**Figure 3.**
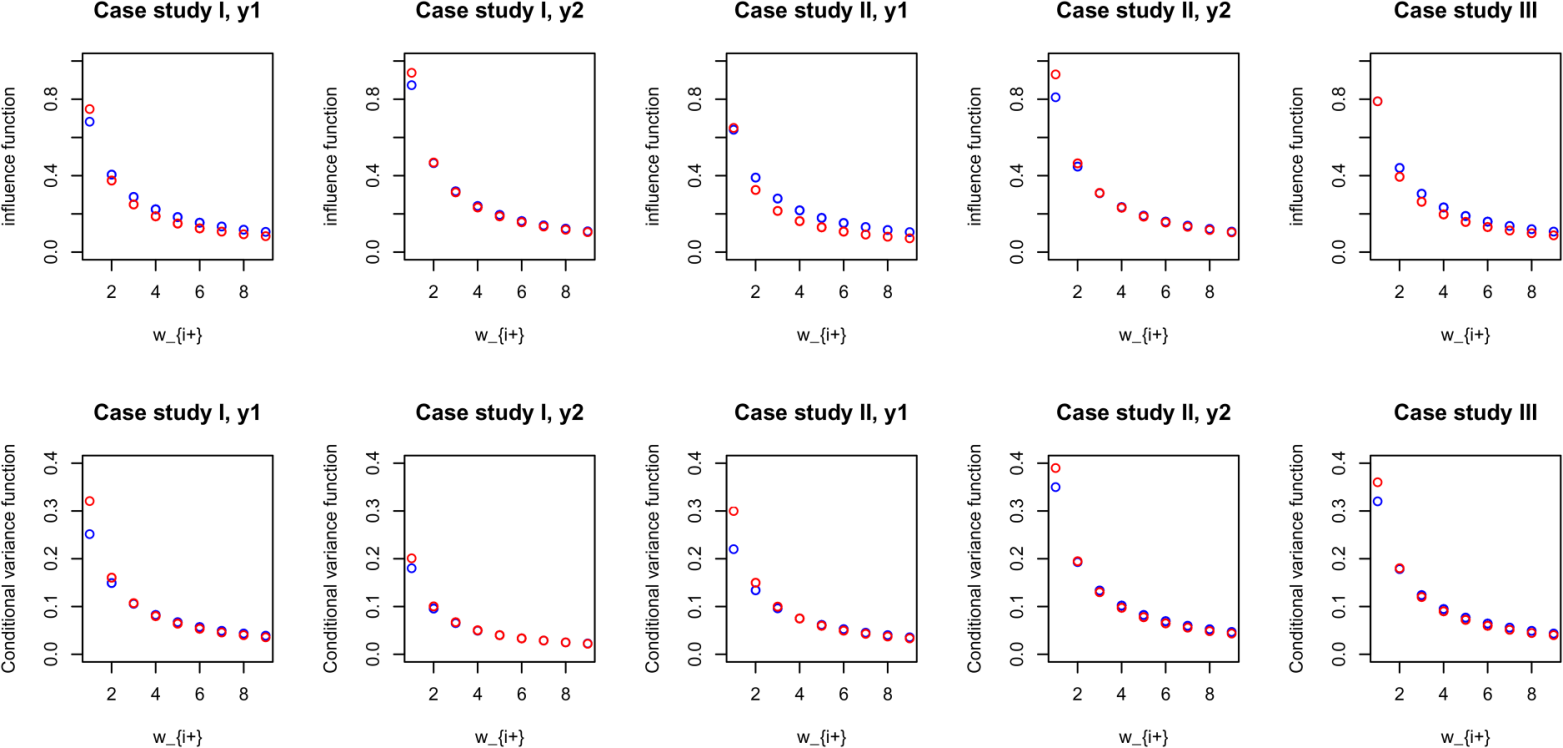
An illustrative comparison of estimated pCAR and LCAR posterior influence and conditional (predictive) variance functions for the three case studies, calculated for the posterior median of parameters c and σ . Red dot: pCAR, Blue dot: LCAR. pCAR: proper conditional autoregressive model; LCAR: Leroux et al. conditional autoregressive model.

Figure 3 illustrates comparable pCAR and LCAR posterior influence and predictive variance functions in the three case studies, calculated for the posterior medians of the spatial and scale parameters.

**Figure 4.**
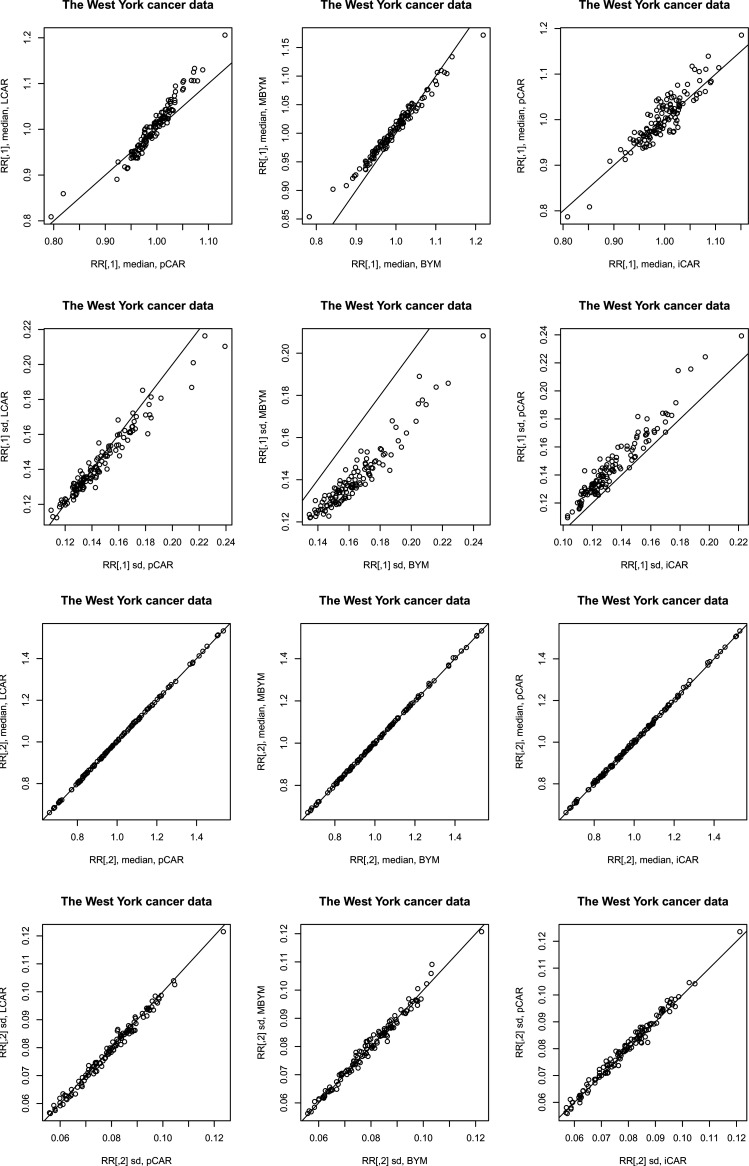
Posterior relative risk predictions for case study II: median – posterior median, sd – posterior standard deviation.

The DIC and WAIC results were overall consistent in each of the three case studies (see the supplement Tables S9 and S10). Of note is the DIC results for $y_{1}$ of the case study II (data of extremely small counts), where comparable DIC scores but modestly different deviance and pD scores were observed among the risk models; similar results were also observed from the associated WAIC scores. Overall, and among the five risk models, posterior risk prediction and inference were nearly identical for our illustrative data of modest or large sample sizes; this is shown in Figure 4 for results of the case study II, as well as the supplement Figures S18 and S19 for results of the case studies I and III. For risk mapping of oral cavity cancer ( $y_{1}$ of case study II), Figure 4 shows the varying degrees of minor or modest posterior risk prediction sensitivities to prior specifications.

**Figure 5.**
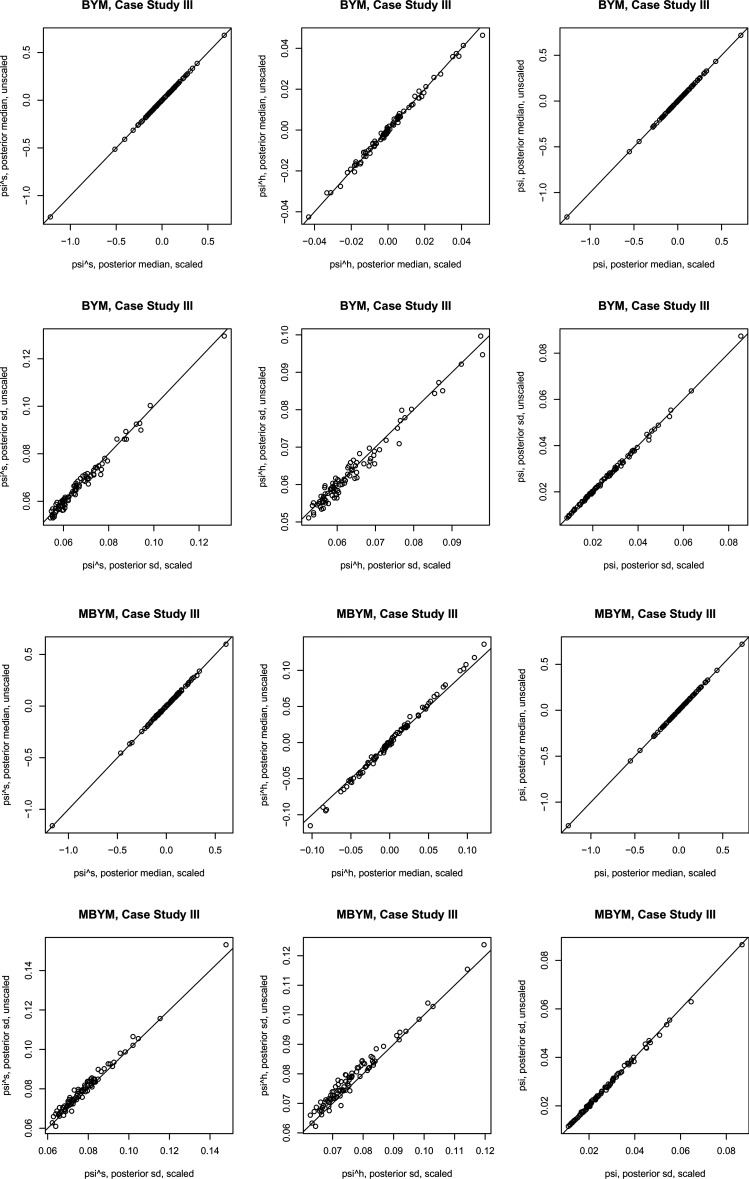
Posterior estimates, posterior median and standard deviation (sd), of the (M)BYM versus scaled (M)BYM components ψ , $\psi^{s}$ and $\psi^{h}$ . The case study III. BYM: Besag, Yorke, and Mollie; MBYM: modified Besag, Yorke, and Mollie.

Results of the scaled iCAR and (M)BYM were also comparable to those of their unscaled counterparts. Figure 5 illustrates the results for (M)BYM of case study III; also see the supplement Tables S9 and S10 for DIC and WAIC results.

### 4.2 Disease risk mapping with covariates: Spatial regression in case study III

We present results of fitting the COVID-19 data to the spatial GLMM (1)–(4) without and with (five) covariates; see Table 6 for the names of the five covariates.

**Table 6.** Posterior estimates, median and standard deviation (sd), of the model parameters without covariate (0 covar.) or with five covariates (5 covar.), for indicated priors. For BYM( $\sigma_{s},\sigma_{h}$ ), $c=\sqrt{\sigma_{s}^{2}/(\sigma_{s}^{2}+\sigma_{h}^{2})}$ , $\sigma =\sqrt{\sigma_{s}^{2}+\sigma_{h}^{2}}$ ; for MBYM( c,σ ), $\sigma_{s}=\sigma \sqrt{c}$ , $\sigma_{h}=\sigma \sqrt{1-c}$ . The five covariates are scores of: Private transportation to work ( $x_{1}$ ), Age 55–64 ( $x_{2}$ ), Education less than high school ( $x_{3}$ ), Colleage education ( $x_{4}$ ), and Unemployment ( $x_{5}$ ). The case study III.

	BYM		BYM		MBYM		MBYM		LCAR		LCAR	
	0 covar.		5 covar.		0 covar.		5 covar.		0 covar.		5 covar.	
Para.	Median	sd	Median	sd	Median	sd	Median	sd	Median	sd	Median	sd
$\beta_{0}$	0.00	0.01	0.00	0.01	0.01	0.01	0.00	0.01	0.01	0.07	0.01	0.04
$\beta_{1}$			0.79	0.68			0.88	0.69			0.81	0.69
$\beta_{2}$			− 4.53	1.04			− 4.67	1.05			− 4.84	1.05
$\beta_{3}$			3.01	0.82			3.44	0.81			3.16	0.81
$\beta_{4}$			1.03	0.68			1.07	0.68			0.98	0.68
$\beta_{5}$			− 2.57	1.67			− 3.32	1.65			− 2.93	1.65
c	0.96	0.09	0.85	0.23	0.84	0.13	0.53	0.22	0.79	0.14	0.51	0.22
σ	0.31	0.04	0.24	0.05	0.27	0.04	0.18	0.03	0.32	0.03	0.24	0.04
$\sigma_{s}$	0.30	0.05	0.22	0.07	0.24	0.05	0.13	0.05				
$\sigma_{h}$	0.06	0.04	0.09	0.04	0.11	0.03	0.12	0.02				
Deviance		923		922		916		917		916		917
pD		90		89		85		84		85		84
DIC		1013		1011		1001		1001		1001		1001
-2 lppd		892		891		889		890		889		889
$2p_{\text{WAIC 2}}$		104		101		88		90		88		90
WAIC		996		992		977		980		977		979

pCAR: proper conditional autoregressive model; LCAR: Leroux et al. conditional autoregressive model; iCAR: intrinsic conditional autoregressive model; BYM: Besag, Yorke, and Mollie; MBYM: modified Besag, Yorke, and Mollie; DIC: deviance information criterion; WAIC: widely applicable information criterion.

Posterior estimates of the model parameters in spatial GLMM (1)–(4) without or with covariates are presented and compared in Table 6 and the supplement Table S11, where LCAR and pCAR outperformed the (scaled) (M)BYM with lower deviance (Dbar), pD and DIC scores. Consistent (but modest) reductions of the CAR spatial and scale parameter estimates from the GLMM without covariate to with covariates were observed (see Table 6 and Table S11), suggesting that the included covariates explained modest amounts of spatial risk dependence and risk variability (see the supplement Figure S20). The (scaled) MBYM, pCAR and LCAR risk models consistently suggested that the unexplained (residual) infection risks might be attributable to omitted covariates of spatially and randomly varying.

**Figure 6.**
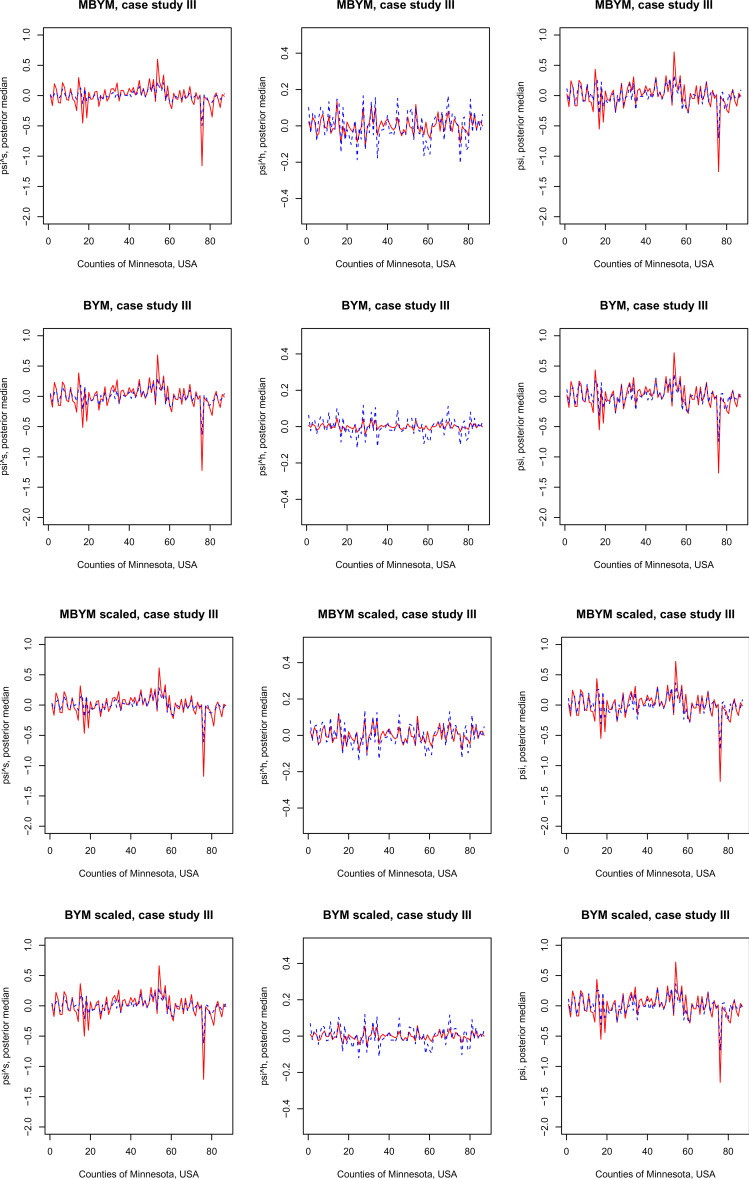
Posterior estimates, posterior median and standard deviation (sd), of the MBYM or BYM components ψ , $\psi^{s}$ and $\psi^{h}$ in the spatial GLMM without and with covariates. Solid (red) line: without covariates, dashed (blue) line: with covariates. The case study III. BYM: Besag, Yorke, and Mollie; MBYM: modified Besag, Yorke, and Mollie; GLMM: generalized linear mixed effects.

Figure 6 presents a comparison of posterior estimates of the (scaled) (M)BYM components of $\psi^{s}$ , $\psi^{h}$ and ψ in GLMM without and with the covariates, showing that the included covariates explained modest amount of variability in the (M)BYM spatial components $\psi^{s}$ (and in ψ ), which is consistent with the results of estimated $\sigma_{s}$ between GLMM without and with covariates.

### 4.3 Adaptive MBYM illustrated in case study III

We illustrate the new proposal of adaptive MBYM( c,σ ), unscaled or scaled MBYM with adaptive spatial weight parameters, for disease mapping without or with covariates. While in this case study the adaptive MBYMs were not shown to outperform their non-adaptive counterparts (in terms of the DIC and WAIC results; see Tables 6 and 7), the purpose of this analysis is to illustrate the potential utility of the adaptive MBYM, which, by allowing for adaptive attributions of spatial and non-spatial components to area-specific (log) relative risks, may inform (and alarm) on neighbourhood risks heterogeneities and clusters.

**Table 7.** Posterior estimates, median and standard deviation (sd), of the adaptive MBYM (unscaled or scaled) model parameters without covariate (0 covar.) or with five covariates (5 covar.). The five covariates are scores of: Private transportation to work ( $x_{1}$ ), Age 55–64 ( $x_{2}$ ), Education less than high school ( $x_{3}$ ), Colleage education ( $x_{4}$ ), and Unemployment ( $x_{5}$ ). The case study III.

	Unscaled		Unscaled		Scaled		Scaled		iCAR		iCAR	
	0 covar.		5 covar.		0 covar.		5 covar.		0 covar.		5 covar.	
Para.	Median	sd	Median	sd	Median	sd	Median	sd	Median	sd	Median	sd
$\beta_{0}$	0.00	0.01	0.00	0.01	0.00	0.01	0.00	0.01	0.00	0.00	0.00	0.00
$\beta_{1}$			0.93	0.70			0.88	0.69			0.73	0.67
$\beta_{2}$			− 4.39	1.01			− 4.12	0.98			− 4.30	1.01
$\beta_{3}$			3.38	0.71			3.11	0.65			2.61	0.68
$\beta_{4}$			0.82	0.63			0.82	0.63			0.97	0.65
$\beta_{5}$			− 2.27	1.55			− 2.90	1.47			− 2.00	1.50
σ	0.21	0.02	0.18	0.02	0.18	0.02	0.15	0.02	0.33	0.03	0.29	0.02
Deviance		925		925		926		925		923		922
pD		94		91		94		93		91		90
DIC		1019		1016		1020		1018		1014		1012
-2lppd		893		893		893		893		892		892
$2p_{\text{WAIC 2}}$		118		113		124		117		107		104
WAIC		1011		1006		1017		1010		1000		996

DIC: deviance information criterion; WAIC: widely applicable information criterion.

Table 7 presents posterior estimates of indicated model parameters, which also includes the results of the non-adaptive iCAR for comparison purpose. The fitted models of adaptive or non-adaptive MBYM consistently suggested that the included covariates explained modest amount of risk variability. The adaptive and non-adaptive MBYMs led to comparable posterior prediction and inference of infection risks.

**Figure 7.**
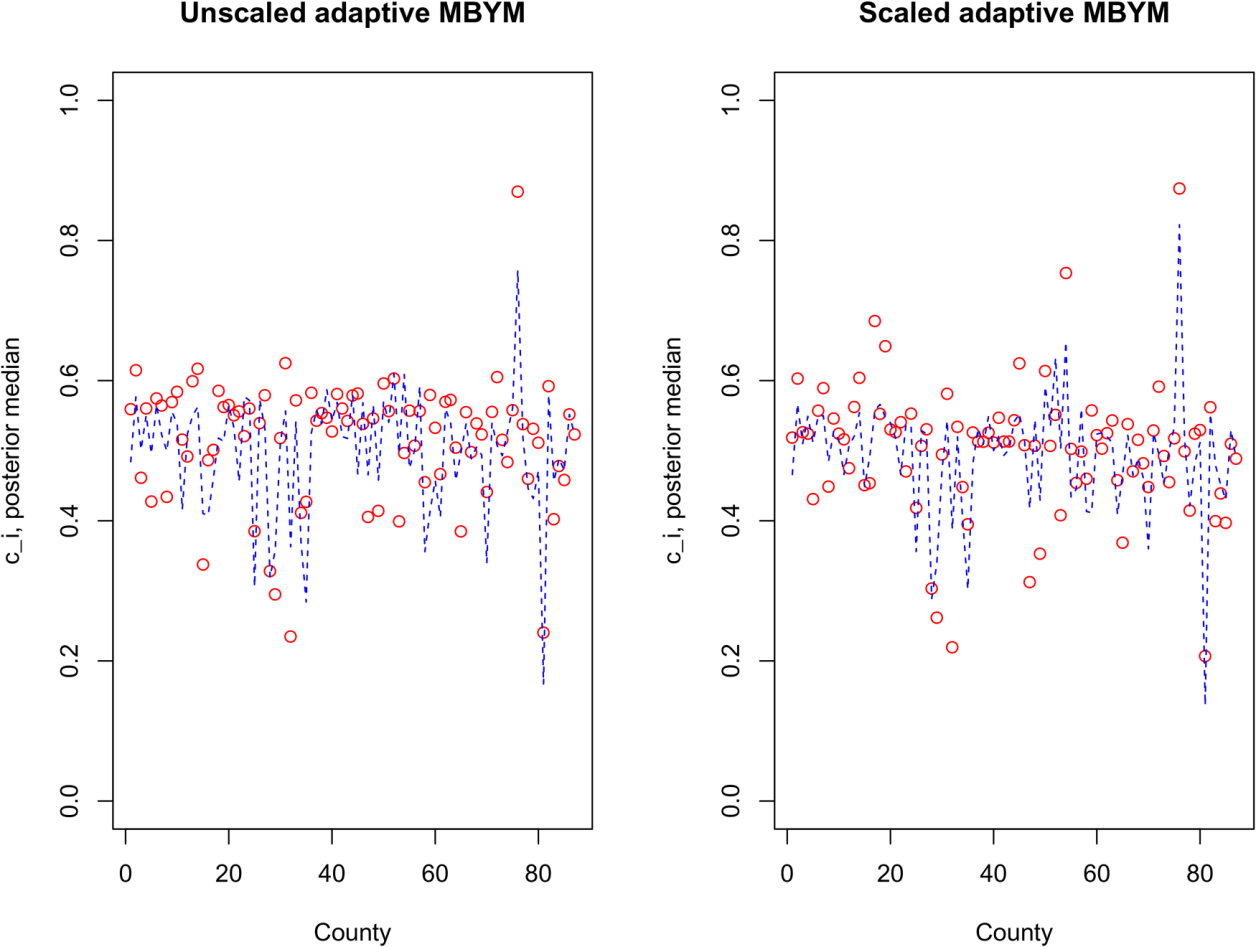
Posterior median of c for indicated adaptive MBYM models without (0 covar.) and with covariates (5 covar.). Dot (red): without covariates, dashed (blue) line: with covariates. The case study III.

Figure 7 illustrates the estimated adaptive spatial parameters, where the scaled and unscaled models led to modestly different but consistent results (e.g. suggesting varied c among the counties). Table 8 illustrates the estimates of $c_{i}$ and relative risks for three counties (named County A, B and C) that had different (estimated) spatial weight parameters. For the model without covariates, for example, County A had a relatively large spatial weight parameter estimate (posterior median = 0.75, sd = 0.23) and a high estimate of infection risk (median = 2.06, sd = 0.03); the County has three neighbours with respective posterior risk estimates that are significantly above 1: (median(sd)) 1.06 (0.03), 1.29 (0.04) and 1.39 (0.04). County C had the lowest posterior estimate of spatial weight parameter (median = 0.21, sd = 0.29), with a relatively low estimate of infection risk (median = 0.76, sd = 0.03); the County has two neighbours that also have low but different infection risk estimates 0.84 (0.01) and 0.28 (0.02). County B, having a relatively high posterior estimate of infection risk (median = 1.37, sd = 0.02) and a spatial weight parameter estimate near 0.5 (median = 0.45, sd = 0.28), has eight neighbours of varying posterior risk estimates; the posterior median ranged between 0.81 (sd = 0.03) and 1.55 (sd = 0.02).

**Table 8.** Posterior estimates, median and standard deviation (sd), of adaptive spatial weight parameters for three illustrative counties, GLMM (adaptive MBYM) without covariate (0 covar.) or with five covariates (5 covar.).

	Adaptive		Adaptive		Non-adaptive		Non-adaptive		
	0 covar.		5 covar.		0 covar.		5 covar.		
Para.	Median	sd	Median	sd	Median	sd	Median	sd	$w_{i+}$
$c_{i}$ , county A	0.75	0.23	0.66	0.26					3
$c_{i}$ , county B	0.45	0.28	0.40	0.27					8
$c_{i}$ , county C	0.21	0.29	0.14	0.29					2
RR, county A	2.06	0.03	2.07	0.03	2.07	0.03	2.07	0.03	3
RR, county B	1.37	0.02	1.37	0.02	1.37	0.01	1.37	0.01	8
RR, county C	0.76	0.03	0.76	0.03	0.75	0.03	0.75	0.03	2

MBYM: modified Besag, Yorke, and Mollie; GLMM: generalized linear mixed effects.

## 5 Summary discussion

This study adds to the Bayesian disease mapping literature in several respects. Analytically and via graphical visualization, we showed that these risk models are Gaussian (Markov) random fields with different spatial dependence (influence) and correlation (covariance) functions. Consequently, they and their multivariate and adaptive model extensions can play different roles in disease mapping applications of contemporary scope and complexity.

Our simulation and case studies, for their scope in illustrating and assessing the iCAR, pCAR, LCAR and (M)BYM risk models together using simulated and real data of extremely small, small, modest and large sample sizes, provided a wealth of important information on the Bayesian posterior estimation, learning, and inference of the model parameters and associated risk prediction and inference, and on the use of DIC and WAIC as tools for evaluations of estimation and out-of-sample predictive models.

In addition, a new proposal of adaptive MBYM is presented and illustrated; it illustrates how the existing spatial risk models can be broadened and extended. We discussed and illustrated the various roles the iCAR, pCAR, LCAR and (M)BYM may play in Bayesian disease mapping, for which we summarize here as takeaway messages.

The pCAR and LCAR are full rank GMRFs that can play nuanced roles of modelling spatial dependence and local influence functions regulated by their respective spatial parameters. The analytic and simulation results favoured LCAR over pCAR when mapping risks of weak or strong spatial correlations. However, pCAR as a spatial model has the advantage for its rich options of multivariate and adaptive generalizations with flexible (multidimensional) spatial dependence and local influence functions.^18,23^ For risk prediction and inference in the context of mapping spatially correlated disease risks, our analytic, simulation and case studies led to consistent results that the two CARs can approximate each other quite well.

The iCAR is a singular GMRF and has an unappealing covariance matrix assuming negative correlations between ‘distance areas’, which may be one reason that it was not favoured as an out-of-sample predictive model in the simulation study. Nevertheless, as an ‘a priori’ spatial smoother, it can be used as a spatial risk prior for modelling spatially structured risk heterogeneity in hierarchical Bayesian models. For the purpose of borrowing information for disease risk mapping, our simulation and case studies suggested that it can be the statistically efficient spatial risk smoother among the five when spatially correlated risks of rare diseases are under study.

The (M)BYMs have dense precision and covariance matrices that postulate practically unappealing but low negative risks dependencies and correlations between ‘distance’ areas. However, they are full rank Gaussian random fields with spatially clustered correlation and partial correlation functions postulating positive risks dependencies and correlations between neighbouring areas. While the utility of (M)BYM for modelling spatial risk dependencies remains a topic of future research, our study suggested evidence that they can be used as (1) estimation and prediction models and (2) as random effects priors for modelling additive components of spatially and randomly varying effects. Compared to fitting the MBYM( c,σ ), a reparameterized BYM with $\sigma_{s}=\sigma \sqrt{c}$ and $\sigma_{h}=\sigma \sqrt{1-c}$ , fitting BYM( $\sigma_{s},\sigma_{h}$ ) via (weakly) information priors on their scale parameters have the advantage that no functional constraints are placed on the BYM scale parameters. The small sample performance of posterior estimation of the MBYM spatial weight parameter c can have a notable impact on the performances of posterior estimation and inference on $\sigma_{s}$ and $\sigma_{h}$ and on the associated components $\psi^{s}$ and $\psi^{s}$ .

Via simulation and case studies, we illustrated that, gaining identifiability via weakly informative prior Uniform(0, a) for the BYM scale parameters or via re-parameterization for MBYM, the BYM and MBYM can facilitate characterization of risk effects ψ as additive spatial and non-spatial components. For this reason, compared to the pCAR and LCAR, which model spatially structure variation in a single set of random effects, the (M)BYM may be a plausible model option in disease mapping without or with covariates. When a regression part is included to explain disease risks variation, the (M)BYM can facilitate assessment of residual risk variation attributable to omitted covariates that are spatially and/or randomly varying.

The new adaptive MBYM is proposed and illustrated for more flexible posterior risk estimation and inference and for unveiling neighbourhood risks clusters and heterogeneities. In a recent study, MacNab^18^ showed, analytically and via a case study, that adaptive extensions of the iCAR, pCAR and LCAR lead to CAR models of different local influence functions; they can be used to model different patterns of locally varying influence functions that characterize local dependencies and spatial discontinuities.

Consistent with the analytic results presented herein and in MacNab,^6,27^ our simulation and case studies also suggested that among the commonly used risk priors none was shown to significantly outperform the others in all disease mapping applications. Noted in MacNab,^6,18^ and suggested by the results of the current study, Bayesian sensitivity analysis with respect to posterior risks prediction and inference, with goodness-of-fit, predictive accuracy, and model complexity assessments such as the DIC and WAIC scores being evaluated and illustrated herein (or model assessment criterions not discussed herein), is still a viable approach for model evaluation, comparison and selection. More importantly, the risk models discussed herein for their nuanced roles in disease mapping can be used as competing or complementary methods for in-depth analysis of disease mapping data.

For data of small or modest sample size, informative hyper-priors for pCAR or LCAR or MBYM spatial parameters can significantly reduce its posterior bias and uncertainty, as illustrated in our simulation and case studies. The present study also showed that both the BYM and MBYM enable (nearly) unbiased posterior estimation of the spatial and non-spatial components $\psi^{s}$ and $\psi^{h}$ , and informative spatial parameter prior for MBYM can reduce posterior risk prediction uncertainties and improve posterior coverage rates of $\psi^{s}$ and $\psi^{h}$ for data of small or modest sample size. A potentially fruitful direction of future research is to further explore and utilize pCAR, LCAR and (M)BYM, and their multivariate/multidimensional and/or adaptive extensions, for Bayesian learning of spatial dependencies, local influences, spatial heterogeneities and discontinuities in the context of (big) rich data analytics and health data science for knowledge learning and discovery concerning spatial epidemiology, population and public health, medicine and beyond.

## Supplemental Material

sj-pdf-1-smm-10.1177_09622802221129040 - Supplemental material for Revisiting Gaussian Markov random fields and Bayesian disease mapping Supplemental material, sj-pdf-1-smm-10.1177_09622802221129040 for Revisiting Gaussian Markov random fields and Bayesian disease mapping by Ying C MacNab in Statistical Methods in Medical Research

## References

[1] Besag J York J Mollie A . Bayesian image restoration, with two applications in spatial statistics. Ann Inst Stat Math 1991; 43: 1–20.

[2] Lawson AB . Bayesian disease mapping hierarchical modeling in spatial epidemiology. (Third Ed.) Chapman and Hall/CRC, 2018.

[3] Martinez-Beneito MA Botella-Rocamora P . Disease mapping: from foundations to multidimensional modeling. CRC Press, 2019.

[4] Cressie N . Statistics for spatial data. (revised ed.) New York: Wiley, 1993.

[5] Leroux BG Lei X Breslow N . Estimation of disease rates in small areas: a new mixed model for spatial dependence. In: Halloran ME and Berry D (eds) *Statistical models in epidemiology, the environment and clinical trials*. Springer, New York, 1999. pp. 135–178.

[6] MacNab YC . On Gaussian Markov random fields and Bayesian disease mapping. Stat Methods Med Res 2011; 20: 49–68. 20547586 10.1177/0962280210371561

[7] Riebler A Sørbye SH Simpson D , et al. An intuitive Bayesian spatial model for disease mapping that accounts for scaling. Stat Methods Med Res 2016; 25: 1145–1165. 27566770 10.1177/0962280216660421

[8] Simpson D Rue H Riebler A , et al. Penalising model component complexity: a principled, practical approach to constructing priors. Stat Sci 2017; 32: 1–28.

[9] Botella-Rocamora P Martinez-Beneito MA Banerjee S . A unifying modeling framework for highly multivariate disease mapping. Stat Med 2015; 34: 1548–1559. 25645551 10.1002/sim.6423

[10] MacNab YC . Linear models of coregionalization for multivariate lattice data: order-dependent and order-free MCARs. Stat Methods Med Res 2016b; 25: 1118–1144. 27566769 10.1177/0962280216660419

[11] MacNab YC . Bayesian estimation of multivariate Gaussian Markov random fields with constraint. Stat Med 2020; 39: 4767–4788. 32935375 10.1002/sim.8752

[12] Martinez-Beneito MA . A general modeling framework for multivariate disease mapping. Biometrika 2013; 100: 539–553.

[13] Gelman A Carlin JB Stern HS , et al. Bayesian data analysis. (Third ed.) Chapman and Hall/CRC, 2014.

[14] Watanabe S Opper M . Asymptotic equivalence of Bayes cross validation and widely applicable information criterion. J Mach Learn Res 2010; 11: 3571–3594.

[15] Watanabe S . A widely applicable information criterion in singular learning theory. J Mach Learn Res 2013; 14: 867–897.

[16] Besag J . Spatial interaction and the statistical analysis of lattice systems (with discussions). J R Stat Soc: Ser B 1974; 36: 192–236.

[17] Rue H Held L . Gaussian Markov random fields - theory and applications. New York: Chapman & Hall, 2005.

[18] MacNab YC . Bayesian disease mapping: past, present, and future. Spat Stat 2022; 50: 100593. 35075407 10.1016/j.spasta.2022.100593 PMC8769562

[19] Corpas-Burgos F Martinez-Beneito MA . On the use of adaptive spatial weight matrices from disease mapping multivariate analyses. Stoch Environ Res Risk Assess 2020; 34: 531–544.

[20] Sun D Tsutakawa RK Speckman PL . Posterior distribution of hierarchical models using CAR(1) distributions. Biometrika 1999; 86: 341–350.

[21] Congdon P . A spatially adaptive conditional autoregressive prior for area health data. Stat Methodol 2008; 5: 1572–3127.

[22] Lee D . A comparison of conditional autoregressive models used in Bayesian disease mapping. Spat Spatiotemporal Epidemiol 2011; 2: 79–89. 22749587 10.1016/j.sste.2011.03.001

[23] MacNab YC . Some recent work on multivariate Gaussian Markov random fields (with discussions). TEST 2018; 27: 497–541.

[24] Elberly LE Carlin BP . Identifiability and convergence issues for Markov chain Monte Carlo fitting of spatial models. Stat Med 2000; 19: 2279–2294. Wiley, New York. 10960853 10.1002/1097-0258(20000915/30)19:17/18<2279::aid-sim569>3.0.co;2-r

[25] Sorbye SH Rue H . Scaling intrinsic Gaussian Markov random field priors in spatial modelling. Spat Stat 2014; 8: 39–51.

[26] Assuncao R Krainski E . Neighborhood dependence in Bayesian spatial models. Biom J 2009; 51: 851–869. 19827056 10.1002/bimj.200900056

[27] MacNab YC . On identification in Bayesian disease mapping and ecological-spatial regression. Stat Methods Med Res 2014; 23: 134–155. 22573502 10.1177/0962280212447152

[28] Jin X Carlin BP Banerjee S . Order-free co-regionalized areal data models with application to multiple-disease mapping. J R Stat Soc: Ser B 2007; 269: 817–838. 10.1111/j.1467-9868.2007.00612.x PMC2963450 20981244

[29] MacNab YC . Linear models of coregionalization for multivariate lattice data: a general framework for coregionalized multivariate CAR models. Stat Med 2016; 35: 3827–3850. 27091685 10.1002/sim.6955

[30] Spiegelhalter D Thomas A Best N , et al. WinBUGS User manual. 2003.

[31] Thomas A Best N Lunn D , et al. GeoBUGS User Manual, 2004.

