# Supplementary material for: Revisiting Gaussian Markov random fields and Bayesian disease mapping
Source: Stat Methods Med Res. 2022 Nov 1;32(1):207–25. doi: 10.1177/09622802221129040 (PMC9814028; doi:10.1177/09622802221129040)
Supplement: sj-pdf-1-smm-10.1177_09622802221129040 - Supplemental material for Revisiting Gaussian Markov random fields and Bayesian disease mapping [file sj-pdf-1-smm-10.1177_09622802221129040.pdf]

Date of last modification - April 19, 2022

# Supplementary Material to the manuscript "Revisiting Gaussian Markov random fields and Bayesian disease mapping"

**Ying C MacNab**

**Abstract** This Supplementary Material for the manuscript "Revisiting Gaussian Markov random fields and Bayesian disease mapping" has three parts. Part I presents two figures that illustrate spatial correlation and variance functions for the pCAR, LCAR, iCAR, and (M)BYM. They are discussed in the main manuscript, where they are named Figures S1 and S2. Part II provides a detailed account on the simulation study, including information on the simulation design (scenarios), a brief outline of the Bayesian implementations, and a full report on results with fifteen figures and ten tables. Part III contains supplementary (eleven) tables and (eighteen) figures illustrating the simulation results presented in Part II and results of the case studies in the manuscript, where they are named Figures S3 - S20 and Tables S1 - S11.

School of Population and Public Health, University of British Columbia, Vancouver, Canada
E-mail: ying.macnab@ubc.ca

# 1 Part I: Spatial correlation and variance functions illustrated

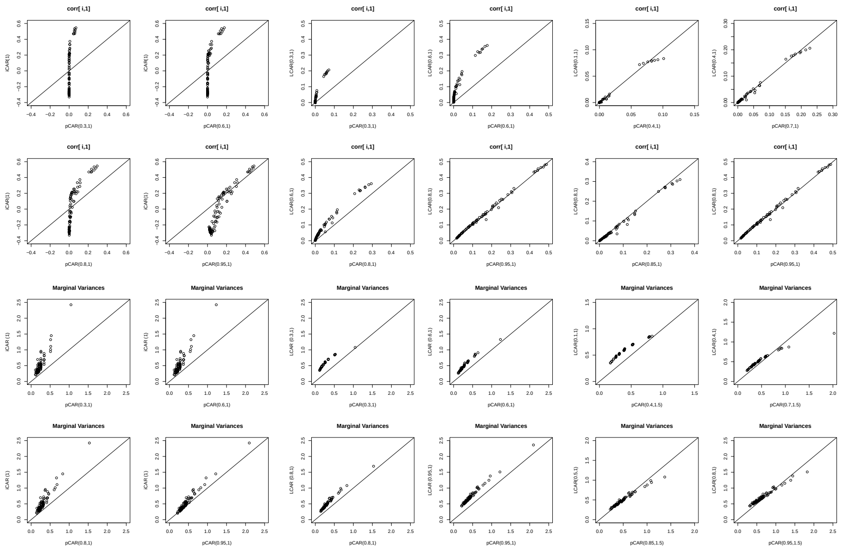

**Fig. 1** The spatial correlation and variance functions are illustrated for the pCAR, LCAR, iCAR, and MBYM, respectively, with indicated parameter values. The spatial correlation functions display correlations between county 1 and county $i$, for all $i \neq 1$. The variance functions display marginal variances of all counties. The Minnesota county map.

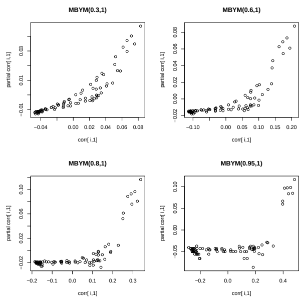

**Fig. 2** Spatially varying or clustered partial and marginal correlation functions are illustrated for MBYM. The (partial) correlation functions display (partial) correlations between county 1 and county $i$, for all $i \neq 1$. The Minnesota county map.

## 2 Part II: The simulation study

We carried out a comprehensive simulation study in the context of hierarchical Bayesian estimation, learning, prediction and inference of GLMM (1)-(4), for options of the non-adaptive CAR or convolution risk models as random effects prior. Posteriors of all unknowns were estimated via Markov chain Monte Carlo (MCMC) simulations implemented in **WinBUGS**, a free software for **B**ayesian Inference **U**sing **G**ibbs **S**ampling in **Win**dows, (Spiegelhalter et al. 2003, MacNab 2011, Lawson 2018, Martinez-Beneito and Botella Rocamora 2019). It is worth mentioning that formulating GMRFs via full conditionals facilitates coding the powerful Gibbs sampler as a computational tool for posterior estimation of the spatial random effects with CAR/GMRF priors via MCMC simulations (Besag et al 1991, Thomas et al 2004).

### 2.1 Simulation scenarios and preliminaries

For the Minnesota county map and its adjacency-defined neighbourhood system, count data were generated for the GLMM (1)-(4) of Poisson likelihood and indicated risk prior options, where the county-level expected counts, $E_i, \forall i$, were the Jin et al (2007) expected counts for previously mentioned cancer of esophagus (named the scenario 1, for a rare disease) or lung (named the scenario 2, for a more common disease) or larynx (named the scenario 3, for a more rare disease). The three scenarios illustrate typical examples of incidence or mortality risk mapping for rare, more common, and more rare diseases, respectively. Scenario 4 serves to illustrate large sample performances; the expected counts were generated from uniform distribution U(5000, 10,000).

WinBUGS programs for MCMC simulations were executed from R via R2WinBUGs function. For the four scenarios, options of the true values of the model parameters that are similar to the estimated GLMM (1)-(4) parameters under indicated prior options for mapping Minnesota cancer mortality of esophagus or lung were considered; see Table S1 (and results for case study I in Section 4).

Simulation results discussed herein are based on 500 simulated data sets generated for each of the indicated models and true parameter values, where the coverage rates are rates of coverage of 95% credible intervals. All results were based on 3,000 posterior samples of the unknowns; the MCMC simulations were implemented for n.chains=3, n.burnin=1,000 and n.thin=10.

We mainly present results of data-driven posterior estimation of unknown parameters, based on commonly used non-informative or weakly informative priors for the fixed effect $\mu$ ($\mu \sim$ dflat()), the spatial parameter $c$ ($c \sim$ dbeta$(1,1)$ or $c \sim$ dunif$(-1,1)$ for pCAR only), and the scale parameter(s) ($\sigma \sim$ dunif$(0,10)$). It is well known that BYM estimation can be sensitive to commonly considered informative hyper-prior options of $\tau_h(\tau_s) \sim \text{Gamma}(a,b)$ (say, the GeoBUGS recommendations, Thomas et al 2004), or weakly informative $\sigma_h(\sigma_s) \sim \text{Uniform}(0,k)$, $k = 5$ or 10. Hyper-prior $\sigma_h(\sigma_s) \sim \text{Uniform}(0,5)$

as a weakly informative prior option led to stable MCMC implementation, for which results of BYM are reported here. For pCAR, LCAR, and MBYM, we also present results of Bayesian sensitive analysis under hyper-prior $c \sim$ dbeta$(3, 6)$ (favouring small $c$) and $c \sim$ dbeta$(8, 3)$ (favouring large $c$).

## 2.2 Posterior estimation and inference of model parameters

Overall, the pCAR, LCAR, and (scaled) MBYM led to comparable performances in terms of posterior estimation of the spatial dependence or weight parameter: Under non-informative prior $c \sim$ Beta$(1, 1)$ for $c \in (0, 1)$, all indicating a tendency of underestimating a large spatial parameter ($c = 0.8$) or over-estimating a small spatial parameter ($c = 0.3$), with considerable posterior uncertainties; Tables S2-S7 illustrate key results under simulation scenarios listed in Table S1, and for the IIDN ($c = 0$) and iCAR ($c = 1$) models. The pCAR under prior $c \sim$ Unif$(-1, 1)$ led to underestimation of both large or small spatial dependence parameters. Posterior biases and uncertainties decreased as the sample size increased (see Tables S2) or when informative priors for $c$ were used; see Tables S3 and S4 for illustrative results of posterior estimates under $c \sim$ Beta$(3, 6)$ (favouring small $c$) and $c \sim$ Beta$(8, 3)$ (favouring large $c$), respectively. Small or modest postesrior biases and comparable performances were observed from posterior estimation of the scale parameter $\sigma$.

Table S5 presents results of fitting the five risk priors to data generated from the GLMM with IIDN risk prior for simulation scenarios 1 and 2. The LCAR resulted in the lowest posterior bias for $c$, with the exception of the pCAR with hyper prior $c \sim$ Unif$(-1, 1)$, which led to the lowest posterior bias for $c$ but the highest posterior bias for $\sigma$.

For all simulation scenarios, the iCAR scale parameter was estimated with near-zero posterior bias and near-the-target coverage rates (see Tables S6).

Consistent and comparable performances were observed from the (M)BYM and scaled (M)BYM risk models; Table S7 illustrates posterior estimation of the model parameters, where $\sigma_s$ and $\sigma_h$ are scale parameters for the spatial and non-spatial components $\boldsymbol{\psi}^s \sim$ iCAR$(\sigma_s)$ or $\boldsymbol{\psi}^s \sim$ scaled iCAR$(\sigma_s)$ and $\boldsymbol{\psi}^h \sim$ IIDN$(\sigma_h)$; $\sigma = \sqrt{\sigma_s^2 + \sigma_h^2}$ and $c = \sigma_s^2/\sigma^2$ for the (scaled) BYM, and $\sigma_s = \sigma\sqrt{c}$ and $\sigma_h = \sigma\sqrt{1-c}$ for (scaled) MBYM$(c, \sigma)$. The (scaled) BYM was shown to perform slightly better than the (scaled) MBYM, observed from modestly lower posterior biases and rmses from the (scaled) BYM; this is particularly evident for data of extremely small sample size (see the results for scenario 3), likely as a result that the spatial weight parameter in MBYM is typically underestimated for a large $c$ and over estimated for a small $c$. For simulation scenarios 1 and 2, the scaled and unscaled (M)BYM led to comparable posterior bias, rmse, coverage rate for all model parameters. Under scenario 3, the performances of the scaled and unscaled (M)BYM differed modestly.

### 2.3 Posterior risks prediction and inference

Overall, with minor or modest differences, the CAR and (M)BYM models led to consistent and comparable performances in terms of posterior risk prediction and inference (see Figures S3-S7). For data of (extremely) small sample size, the pCAR and LCAR had modestly lower posterior risk biases and standard deviations, compared to their (M)BYM counterparts. For all simulation scenarios, the iCAR performed well in terms of posterior risk prediction and inference (see. Fig. S7). For all models and simulation scenarios, and even for extremely small sample size (the simulation scenario 3), the 95% credible intervals for the county-specific relative risks led to near or above 90% coverage rate.

Figures S8 and S9 present results on sensitivities of posterior risk prediction and inference to prior (mis)specifications, where the x-axis presents results of the true risk model, while the y-axis displays results of the fitted risk model. Minor or modest prior sensitivities were observed, most notably in the posterior risk standard deviations, which suggests that as spatial smoothers the five risk models can approximate each other; this is also suggested by the resulting DIC and WAIC statistics (e.g. mean scores and associated standard deviations as illustrated in Tables 2 and 3 the manuscript).

For pCAR, LCAR, and MBYM, minor or modest posterior risk sensitivities to spatial parameter prior options were mostly observed from the posterior risk standard deviations (posterior risk uncertainties) and resulting posterior risk coverage rates; posterior risk bias and root mean square errors remained robust; Figure S10 illustrates the results, where informative spatial parameter priors led to reduced posterior risk standard deviations and improved posterior risk coverage rates.

Fig. S11 illustrates comparable performances of the BYM and MBYM in terms of posterior prediction and inference for relative risks (RR) and the components $\boldsymbol{\psi}^s$ and $\boldsymbol{\psi}^h$, respectively, with the exception of scenario 3 (see S3(c=0.8) or S3(c=0.3) in Fig. S11), for which MBYM was shown to have consistently and notably higher or lower than 0.95 coverage rates for $\boldsymbol{\psi}^s$, as a result that the non-informative hyper-prior $c \sim \text{Beta}(1,1)$ led to high posterior bias for $c$ (i.e. as a result of over- or under-estimated spatial parameter). Informative spatial parameter priors led to reduced posterior standard deviations (posterior uncertainties) and improved posterior coverage rates for the respective MBYM components $\boldsymbol{\psi}^s$ and $\boldsymbol{\psi}^h$, as the Fig. S12 illustrates.

## 3 Part III: Figures and Tables for the simulation and case studies

**Table 1** The design of the simulation study.

| Sceonario | Expected counts | True values of the parameters |
|---|---|---|
| | (min, median, max) | $\boldsymbol{\theta} = (c, \sigma, \mu)$ |
| Sceonario 1a | (2.4, 11.2, 418.5) | $\boldsymbol{\theta} = (0.6, 0.35, -0.05)$ |
| Sceonario 2a | (28.7, 131.7, 4901.3) | $\boldsymbol{\theta} = (0.8, 0.25, -0.07)$ |
| Sceonario 1b | (2.4, 11.2, 418.5) | $\boldsymbol{\theta} = (0.3, 0.35, -0.05)$ |
| Sceonario 2b | (28.7, 131.7, 4901.3) | $\boldsymbol{\theta} = (0.3, 0.35, -0.07)$ |
| Sceonario 1c | (2.4, 11.2, 418.5) | IIDN, $\boldsymbol{\theta} = (0, 0.35, -0.05)$ |
| Sceonario 2c | (28.7, 131.7, 4901.3) | IIDN, $\boldsymbol{\theta} = (0, 0.35, -0.07)$ |
| Sceonario 1d | (2.4, 11.2, 418.5) | iCAR, $\boldsymbol{\theta} = (1, 0.35, -0.05)$ |
| Sceonario 2d | (28.7, 131.7, 4901.3) | iCAR, $\boldsymbol{\theta} = (1, 0.35, -0.07)$ |
| Sceonario 3a | (0.62, 2.8, 105.5) | $\boldsymbol{\theta} = (0.6, 0.35, -0.05)$ |
| Sceonario 3b | (0.62, 2.8, 105.5) | $\boldsymbol{\theta} = (0.8, 0.25, -0.07)$ |
| Sceonario 3c | (0.62, 2.8, 105.5) | $\boldsymbol{\theta} = (0.3, 0.25, -0.07)$ |
| Sceonario 4a | U(5000, 10,000) | $\boldsymbol{\theta} = (0.3, 0.35, -0.05)$ |
| Sceonario 4b | U(5000, 10,000) | $\boldsymbol{\theta} = (0.8, 0.35, -0.05)$ |

**Table 2** Summary results of the simulation study for posterior estimation of the indicated model parameters and simulation scenarios. mean: mean of the posterior mean, rmse: square-root of mean square errors, cover rate: 95% credible interval coverage rate. †: $c_k \sim$ Beta(1, 1) k=1,2; ††: $c_k \sim$ Unif$(-1, 1), k = 1, 2$.

| Node | True value | mean rmse rate | mean rmse rate | mean rmse rate | mean rmse rate |
|---|---|---|---|---|---|
| | | pCAR† | pCAR†† | LCAR | MBYM |
| Scenarios 1a and 2a | | | | | |
| $c_1$ | 0.60 | 0.54 0.15 1.00 | 0.10 0.55 1.00 | 0.57 0.14 1.00 | 0.60 0.15 0.99 |
| $c_2$ | 0.80 | 0.69 0.21 0.97 | 0.56 0.34 0.93 | 0.69 0.18 0.98 | 0.74 0.15 0.97 |
| $\sigma_1$ | 0.35 | 0.34 0.09 0.94 | 0.34 0.09 0.94 | 0.33 0.08 0.95 | 0.37 0.06 0.97 |
| $\sigma_2$ | 0.25 | 0.26 0.03 0.95 | 0.26 0.03 0.95 | 0.24 0.03 0.94 | 0.25 0.04 0.97 |
| $\mu_1$ | -0.05 | -0.05 0.04 0.97 | -0.05 0.04 0.94 | -0.06 0.07 0.96 | -0.05 0.04 0.96 |
| $\mu_2$ | -0.07 | -0.07 0.03 0.93 | -0.07 0.03 0.94 | -0.07 0.07 0.86 | -0.07 0.02 0.94 |
| Scenarios 1b and 2b | | | | | |
| $c_1$ | 0.30 | 0.49 0.24 0.99 | 0.10 0.42 0.99 | 0.46 0.23 0.97 | 0.42 0.18 0.98 |
| $c_2$ | 0.30 | 0.39 0.21 0.96 | 0.25 0.36 0.91 | 0.41 0.22 0.89 | 0.38 0.15 0.97 |
| $\sigma_1$ | 0.35 | 0.33 0.10 0.94 | 0.33 0.10 0.94 | 0.36 0.08 0.95 | 0.38 0.07 0.96 |
| $\sigma_2$ | 0.35 | 0.35 0.04 0.93 | 0.34 0.04 0.93 | 0.37 0.06 0.91 | 0.37 0.05 0.96 |
| $\mu_1$ | -0.05 | -0.05 0.04 0.96 | -0.05 0.04 0.95 | -0.05 0.06 0.98 | -0.05 0.05 0.93 |
| $\mu_2$ | -0.07 | -0.07 0.02 0.97 | -0.07 0.02 0.97 | -0.07 0.05 0.94 | -0.07 0.03 0.95 |
| Scenarios 3a and 3b | | | | | |
| $c_1$ | 0.60 | 0.53 0.09 1.00 | 0.07 0.57 1.00 | 0.55 0.10 1.00 | 0.54 0.13 1.00 |
| $c_2$ | 0.80 | 0.54 0.27 1.00 | 0.07 0.75 0.99 | 0.55 0.26 1.00 | 0.56 0.26 1.00 |
| $\sigma_1$ | 0.35 | 0.36 0.14 0.98 | 0.36 0.14 0.98 | 0.33 0.12 0.98 | 0.35 0.11 0.98 |
| $\sigma_2$ | 0.25 | 0.30 0.13 0.98 | 0.30 0.14 0.98 | 0.25 0.11 0.98 | 0.24 0.09 0.96 |
| $\mu_1$ | -0.05 | -0.06 0.06 0.96 | -0.06 0.06 0.94 | -0.06 0.08 0.96 | -0.06 0.06 0.98 |
| $\mu_2$ | -0.07 | -0.08 0.06 0.95 | -0.08 0.06 0.94 | -0.07 0.08 0.93 | -0.08 0.06 0.96 |
| Scenarios 4a and 4b | | | | | |
| $c_1$ | 0.30 | 0.36 0.19 0.92 | 0.25 0.20 0.85 | 0.38 0.30 0.84 | 0.37 0.15 0.97 |
| $c_2$ | 0.80 | 0.72 0.17 0.95 | 0.71 0.16 0.95 | 0.72 0.18 0.95 | 0.71 0.17 0.96 |
| $\sigma_1$ | 0.35 | 0.35 0.03 0.95 | 0.36 0.05 0.91 | 0.35 0.03 0.96 | 0.37 0.04 0.96 |
| $\sigma_2$ | 0.35 | 0.35 0.03 0.91 | 0.34 0.04 0.92 | 0.35 0.03 0.92 | 0.35 0.04 0.97 |
| $\mu_1$ | -0.05 | -0.05 0.03 0.95 | -0.05 0.05 0.90 | -0.05 0.02 0.94 | -0.05 0.03 0.95 |
| $\mu_2$ | -0.05 | -0.05 0.04 0.88 | -0.05 0.09 0.78 | -0.05 0.04 0.88 | -0.05 0.03 0.81 |

**Table 3** Summary results of the sensitivity study for posterior estimation of the spatial parameter ($c \in (0,1)$) under the indicated informative or non-informative hyper-prior and for the indicated simulation scenarios. The true model parameters are $\mu = 0.07$, $\sigma = 0.35$, and $c = 0.3$ for scenarios 1(a), 2(a), and 3(a) (denoted S1(a), S2(a), and S3(a)) or $c = 0.8$ for scenarios 1(b), 2(b), and 3(b) (denoted S1(b), S2(b), and S3(b)). mean: mean of the posterior mean, rmse: (square-)root of mean square errors, cover rate: 95% credible interval coverage rate.

| | mean rmse rate | mean rmse rate | mean rmse rate | mean rmse rate | mean rmse rate | mean rmse rate |
|---|---|---|---|---|---|---|
| | pCAR | pCAR | LCAR | LCAR | MBYM | MBYM |
| | $c \sim$ Beta(3, 6) | $c \sim$ Beta(1, 1) | $c \sim$ Beta(3, 6) | $c \sim$ Beta(1, 1) | $c \sim$ Beta(3, 6) | $c \sim$ Beta(1, 1) |
| S1(a) | 0.34 0.05 1.00 | 0.50 0.22 1.00 | 0.35 0.09 1.00 | 0.48 0.24 0.95 | 0.34 0.06 0.96 | 0.42 0.18 0.98 |
| S2(a) | 0.33 0.08 1.00 | 0.40 0.19 0.97 | 0.33 0.10 0.97 | 0.41 0.22 0.89 | 0.33 0.07 1.00 | 0.38 0.15 0.97 |
| S3(a) | 0.34 0.04 1.00 | 0.53 0.23 1.00 | 0.35 0.06 1.00 | 0.52 0.24 1.00 | 0.34 0.05 1.00 | 0.49 0.22 0.99 |
| | $c \sim$ Beta(8, 3) | $c \sim$ Beta(1, 1) | $c \sim$ Beta(8, 3) | $c \sim$ Beta(1, 1) | $c \sim$ Beta(8, 3) | $c \sim$ Beta(1, 1) |
| S1(b) | 0.73 0.07 1.00 | 0.58 0.25 1.00 | 0.73 0.07 1.00 | 0.63 0.22 0.99 | 0.74 0.07 0.97 | 0.64 0.22 0.98 |
| S2(b) | 0.74 0.09 1.00 | 0.67 0.21 0.95 | 0.74 0.08 1.00 | 0.69 0.18 0.98 | 0.75 0.08 1.00 | 0.69 0.18 0.97 |
| S3(b) | 0.73 0.08 1.00 | 0.55 0.26 1.00 | 0.73 0.07 1.00 | 0.57 0.24 1.00 | 0.74 0.07 1.00 | 0.58 0.24 1.00 |

**Table 4** Simulation results for posterior estimation of the (M)BYM scale parameters $\sigma_s$ and $\sigma_h$ for scenarios 1-3 with indicated true parameters ($\mu = 0.07$ and $\sigma = 0.35$ are the true parameters for (M)BYM. mean: mean of the posterior mean, rmse: square-root of mean square errors, cover rate: 95% credible interval coverage rate. MBYM1: MBYM estimated under non-information prior $c \sim \text{Beta}(1,1)$; MBYM2: MBYM estimated under information prior $c \sim \text{Beta}(3,6)$ for true $c = 0.3$ or $c \sim \text{Beta}(8,3)$ for true $c = 0.8$.

| Node | True value | mean rmse rate | mean rmse rate | mean rmse rate |
|---|---|---|---|---|
| Scenarios 1 | | | | |
| | | BYM | MBYM1 | MBYM2 |
| $\sigma_s[1]$ | 0.19 | 0.24 0.11 0.97 | 0.24 0.09 0.98 | 0.21 0.04 1.00 |
| $\sigma_s[2]$ | 0.31 | 0.30 0.09 0.99 | 0.27 0.09 0.97 | 0.30 0.05 0.98 |
| $\sigma_h[1]$ | 0.29 | 0.27 0.06 0.96 | 0.27 0.05 0.96 | 0.29 0.04 0.97 |
| $\sigma_h[2]$ | 0.16 | 0.16 0.05 0.99 | 0.18 0.05 0.98 | 0.17 0.03 0.99 |
| Scenario 2 | | | | |
| | | BYM | MBYM1 | MBYM2 |
| $\sigma_s[1]$ | 0.19 | 0.22 0.08 0.97 | 0.23 0.07 0.97 | 0.21 0.03 1.00 |
| $\sigma_s[2]$ | 0.31 | 0.31 0.07 0.97 | 0.28 0.07 0.96 | 0.30 0.04 0.99 |
| $\sigma_h[1]$ | 0.29 | 0.28 0.04 0.96 | 0.28 0.03 0.95 | 0.29 0.03 0.95 |
| $\sigma_h[2]$ | 0.16 | 0.16 0.04 0.97 | 0.17 0.03 0.95 | 0.17 0.02 0.98 |
| Scenario 3 | | | | |
| | | BYM | MBYM1 | MBYM2 |
| $\sigma_s[1]$ | 0.19 | 0.29 0.14 0.98 | 0.26 0.11 0.99 | 0.20 0.06 0.99 |
| $\sigma_s[2]$ | 0.31 | 0.31 0.11 0.99 | 0.25 0.12 0.95 | 0.30 0.10 0.96 |
| $\sigma_h[1]$ | 0.29 | 0.25 0.09 0.98 | 0.25 0.09 0.94 | 0.28 0.08 0.96 |
| $\sigma_h[2]$ | 0.16 | 0.18 0.06 1.00 | 0.18 0.07 1.00 | 0.17 0.06 0.99 |

**Table 5** Summary results of the simulation study for posterior estimation of the indicated model parameters and simulation scenarios 1c and 2c. mean: mean of the posterior mean, rmse: square-root of mean square errors, cover rate: 95% credible interval coverage rate. †: $c \sim \text{Beta}(1, 1)$, ††: $c \sim \text{Uniform}(-1, 1)$.

| Node | True value | mean rmse | mean rmse | mean rmse | mean rmse | mean rmse rate |
|---|---|---|---|---|---|---|
| Scenario 1c | | | | | | |
| | | LCAR | pCAR† | pCAR †† | MBYM | BYM |
| $c$ | 0.00 | 0.21 0.25 0.00 | 0.35 0.38 0.00 | -0.02 0.36 0.95 | 0.27 0.30 0.00 | 0.31 0.33 0.00 |
| $\sigma$ | 0.35 | 0.45 0.13 0.76 | 0.77 0.43 0.01 | 0.76 0.42 0.01 | 0.41 0.08 0.89 | 0.41 0.08 0.87 |
| $\mu$ | -0.07 | -0.07 0.05 0.99 | -0.07 0.05 0.98 | -0.07 0.04 0.96 | -0.07 0.05 0.95 | -0.07 0.05 0.94 |
| Scenario 2c | | | | | | |
| $c$ | 0.00 | 0.09 0.12 0.00 | 0.24 0.27 0.00 | -0.04 0.33 0.90 | 0.17 0.20 0.00 | 0.23 0.24 0.00 |
| $\sigma$ | 0.35 | 0.41 0.07 0.82 | 0.77 0.42 0.00 | 0.77 0.42 0.00 | 0.38 0.05 0.93 | 0.39 0.05 0.92 |
| $\mu$ | -0.07 | -0.07 0.04 0.96 | -0.07 0.04 0.95 | -0.07 0.03 0.95 | -0.07 0.04 0.92 | -0.07 0.04 0.92 |

**Table 6** Summary results of the simulation study for posterior estimation of the indicated model parameters and simulation scenarios. mean: mean of the posterior mean, rmse: square-root of mean square errors, cover rate: 95% credible interval coverage rate. †: $c_k \sim \text{Beta}(1, 1)$ k=1,2,3, for simulation scenarios 1d, 2d, and 3d.

| Node | True value | mean rmse | mean rmse | mean rmse | mean rmse | mean rmse rate |
|---|---|---|---|---|---|---|
| | | pCAR | LCAR | MBYM | BYM | iCAR |
| $c_1$† | 1.00 | 0.76 0.27 | 0.70 0.32 | 0.74 0.29 | 0.82 0.23 | |
| $c_2$† | 1.00 | 0.86 0.17 | 0.78 0.25 | 0.84 0.19 | 0.90 0.14 | |
| $c_3$† | 1.00 | 0.62 0.40 | 0.61 0.40 | 0.62 0.39 | 0.70 0.33 | |
| $\sigma_1$ | 0.35 | 0.43 0.12 | 0.35 0.07 | 0.30 0.08 | 0.33 0.09 | 0.35 0.07 0.96 |
| $\sigma_2$ | 0.25 | 0.28 0.04 | 0.24 0.03 | 0.22 0.04 | 0.23 0.05 | 0.25 0.03 0.96 |
| $\sigma_3$ | 0.35 | 0.46 0.20 | 0.35 0.13 | 0.30 0.12 | 0.36 0.12 | 0.35 0.13 0.96 |
| $\mu_1$ | -0.05 | -0.05 0.03 | -0.05 0.03 | -0.05 0.03 | -0.05 0.03 | -0.05 0.03 0.94 |
| $\mu_2$ | -0.07 | -0.07 0.01 | -0.07 0.01 | -0.07 0.01 | -0.07 0.01 | -0.07 0.01 0.93 |
| $\mu_1$ | -0.05 | -0.06 0.06 | -0.06 0.06 | -0.06 0.06 | -0.07 0.06 | -0.05 0.06 0.94 |
| $\sigma_1$ | 0.8 | | | | | 0.80 0.10 0.95 |
| $\sigma_2$ | 0.8 | | | | | 0.80 0.07 0.96 |
| $\sigma_3$ | 0.8 | | | | | 0.81 0.16 0.95 |
| $\sigma_1$ | 1.5 | | | | | 1.50 0.14 0.98 |
| $\sigma_2$ | 1.5 | | | | | 1.49 0.12 0.98 |
| $\sigma_3$ | 1.5 | | | | | 1.52 0.21 0.97 |

**Table 7** Summary results of the posterior estimation of the GLMM(1)-(4) model parameters, with (M)BYM or scaled (M)BYM as risk prior and for simulation scenarios 1-3 and indicated true model parameters. mean: mean of the posterior mean, rmse: (square-)root of mean square errors, cover rate: 95% credible interval coverage rate.

| Param | True value | mean | rmse | rate | mean | rmse | rate | mean | rmse | rate | mean | rmse | rate |
|---|---|---|---|---|---|---|---|---|---|---|---|---|---|
| | | BYM | | | scaled BYM | | | MBYM | | | scaled MBYM | | |
| Scenario 1 | | | | | | | | | | | | | |
| $c$ | 0.30 | 0.41 | 0.21 | 0.98 | 0.38 | 0.20 | 0.98 | 0.42 | 0.18 | 0.98 | 0.40 | 0.17 | 0.98 |
| $\sigma$ | 0.35 | 0.40 | 0.08 | 0.94 | 0.36 | 0.05 | 0.95 | 0.38 | 0.07 | 0.95 | 0.36 | 0.05 | 0.96 |
| $\sigma_s$ | 0.19 | 0.24 | 0.11 | 0.97 | 0.20 | 0.07 | 0.98 | 0.24 | 0.09 | 0.98 | 0.21 | 0.06 | 0.98 |
| $\sigma_h$ | 0.29 | 0.29 | 0.06 | 0.96 | 0.27 | 0.04 | 0.96 | 0.27 | 0.05 | 0.96 | 0.27 | 0.06 | 0.96 |
| $\mu$ | -0.07 | -0.07 | 0.04 | 0.96 | -0.07 | 0.05 | 0.95 | -0.07 | 0.04 | 0.96 | -0.07 | 0.05 | 0.94 |
| $c$ | 0.80 | 0.68 | 0.21 | 1.00 | 0.70 | 0.20 | 0.99 | 0.64 | 0.22 | 0.98 | 0.66 | 0.20 | 0.98 |
| $\sigma$ | 0.35 | 0.36 | 0.07 | 0.98 | 0.36 | 0.05 | 0.96 | 0.34 | 0.06 | 0.96 | 0.35 | 0.05 | 0.96 |
| $\sigma_s$ | 0.31 | 0.30 | 0.09 | 0.99 | 0.30 | 0.07 | 0.98 | 0.27 | 0.09 | 0.97 | 0.28 | 0.07 | 0.97 |
| $\sigma_h$ | 0.16 | 0.15 | 0.05 | 0.99 | 0.17 | 0.06 | 0.99 | 0.18 | 0.05 | 0.98 | 0.18 | 0.05 | 0.97 |
| $\mu$ | -0.07 | -0.07 | 0.04 | 0.95 | -0.07 | 0.04 | 0.96 | -0.07 | 0.04 | 0.96 | -0.07 | 0.04 | 0.96 |
| Scenario 2 | | | | | | | | | | | | | |
| $c$ | 0.30 | 0.36 | 0.17 | 0.97 | 0.33 | 0.17 | 0.97 | 0.38 | 0.15 | 0.97 | 0.35 | 0.14 | 0.98 |
| $\sigma$ | 0.35 | 0.38 | 0.05 | 0.96 | 0.36 | 0.04 | 0.92 | 0.37 | 0.05 | 0.96 | 0.36 | 0.03 | 0.94 |
| $\sigma_s$ | 0.19 | 0.22 | 0.08 | 0.97 | 0.19 | 0.07 | 0.97 | 0.23 | 0.07 | 0.97 | 0.20 | 0.05 | 0.98 |
| $\sigma_h$ | 0.29 | 0.28 | 0.04 | 0.96 | 0.29 | 0.04 | 0.96 | 0.28 | 0.03 | 0.95 | 0.28 | 0.04 | 0.96 |
| $\mu$ | -0.07 | -0.07 | 0.03 | 0.95 | -0.07 | 0.03 | 0.94 | -0.07 | 0.03 | 0.95 | -0.07 | 0.03 | 0.93 |
| $c$ | 0.80 | 0.73 | 0.17 | 0.97 | 0.73 | 0.16 | 0.99 | 0.69 | 0.18 | 0.97 | 0.69 | 0.16 | 0.97 |
| $\sigma$ | 0.35 | 0.36 | 0.05 | 0.97 | 0.35 | 0.03 | 0.97 | 0.34 | 0.05 | 0.96 | 0.35 | 0.03 | 0.94 |
| $\sigma_s$ | 0.31 | 0.31 | 0.07 | 0.97 | 0.31 | 0.05 | 0.98 | 0.28 | 0.07 | 0.96 | 0.29 | 0.05 | 0.96 |
| $\sigma_h$ | 0.16 | 0.16 | 0.04 | 0.97 | 0.16 | 0.04 | 0.99 | 0.17 | 0.03 | 0.95 | 0.18 | 0.04 | 0.97 |
| $\mu$ | -0.07 | -0.07 | 0.02 | 0.96 | -0.07 | 0.02 | 0.95 | -0.07 | 0.02 | 0.96 | -0.07 | 0.03 | 0.97 |
| Scenario 3 | | | | | | | | | | | | | |
| $c$ | 0.30 | 0.52 | 0.27 | 0.99 | 0.42 | 0.20 | 1.00 | 0.49 | 0.22 | 0.99 | 0.44 | 0.18 | 1.00 |
| $\sigma$ | 0.35 | 0.42 | 0.13 | 0.93 | 0.35 | 0.08 | 0.97 | 0.38 | 0.11 | 0.95 | 0.32 | 0.10 | 0.94 |
| $\sigma_s$ | 0.19 | 0.29 | 0.14 | 0.98 | 0.20 | 0.07 | 1.00 | 0.26 | 0.11 | 0.99 | 0.20 | 0.06 | 1.00 |
| $\sigma_h$ | 0.29 | 0.25 | 0.09 | 0.98 | 0.25 | 0.09 | 0.97 | 0.25 | 0.09 | 0.94 | 0.23 | 0.10 | 0.92 |
| $\mu$ | -0.07 | -0.09 | 0.07 | 0.94 | -0.09 | 0.07 | 0.94 | -0.08 | 0.07 | 0.94 | -0.08 | 0.07 | 0.94 |
| $c$ | 0.80 | 0.65 | 0.21 | 1.00 | 0.55 | 0.29 | 1.00 | 0.58 | 0.25 | 1.00 | 0.53 | 0.29 | 1.00 |
| $\sigma$ | 0.35 | 0.38 | 0.11 | 0.96 | 0.30 | 0.09 | 0.94 | 0.33 | 0.12 | 0.94 | 0.27 | 0.12 | 0.83 |
| $\sigma_s$ | 0.31 | 0.31 | 0.11 | 0.99 | 0.21 | 0.09 | 0.90 | 0.25 | 0.12 | 0.95 | 0.19 | 0.14 | 0.77 |
| $\sigma_h$ | 0.16 | 0.18 | 0.06 | 1.00 | 0.18 | 0.06 | 1.00 | 0.18 | 0.07 | 1.00 | 0.17 | 0.06 | 1.00 |
| $\mu$ | -0.07 | -0.09 | 0.07 | 0.94 | -0.09 | 0.07 | 0.94 | -0.08 | 0.07 | 0.95 | -0.08 | 0.07 | 0.94 |

**Table 8** Posterior estimation of the indicated model parameters, for all case studies. median: posterior median, sd: posterior standard deviation. †: $c \sim \text{Beta}(1,1)$ for all case studies.

| Node | median sd | median sd | median sd | median sd | median sd |
|---|---|---|---|---|---|
| Case Study I | | | | | |
| | iCAR | pCAR | LCAR | BYM | MBYM |
| $c_1$† | | 0.75 0.25 | 0.69 0.23 | 0.87 0.27 | 0.70 0.04 |
| $c_2$† | | 0.94 0.06 | 0.87 0.12 | 0.98 0.05 | 0.95 0.01 |
| $\sigma_1$ | 0.24 0.09 | 0.32 0.10 | 0.25 0.08 | 0.24 0.08 | 0.20 0.07 |
| $\sigma_2$ | 0.17 0.03 | 0.20 0.03 | 0.18 0.02 | 0.17 0.02 | 0.17 0.03 |
| $\mu_1$ | -0.06 0.03 | -0.05 0.05 | -0.06 0.07 | -0.06 0.04 | -0.05 0.04 |
| $\mu_2$ | -0.08 0.01 | -0.07 0.04 | -0.07 0.05 | -0.08 0.01 | -0.08 0.01 |
| Case Study II | | | | | |
| $c_1$† | | 0.65 0.28 | 0.64 0.27 | 0.81 0.33 | 0.60 0.29 |
| $c_2$† | | 0.93 0.07 | 0.81 0.14 | 0.93 0.08 | 0.88 0.09 |
| $\sigma_1$ | 0.23 0.12 | 0.30 0.14 | 0.22 0.11 | 0.23 0.10 | 0.17 0.10 |
| $\sigma_2$ | 0.37 0.03 | 0.39 0.03 | 0.35 0.03 | 0.33 0.04 | 0.31 0.04 |
| $\mu_1$ | -0.01 0.04 | 0.00 0.04 | -0.01 0.06 | -0.01 0.04 | -0.01 0.04 |
| $\mu_2$ | -0.02 0.01 | -0.02 0.05 | -0.03 0.08 | -0.02 0.01 | -0.02 0.01 |
| Case Study III | | | | | |
| $c$† | | 0.79 0.12 | 0.79 0.14 | 0.97 0.08 | 0.90 0.09 |
| $\sigma$ | 0.34 0.03 | 0.36 0.03 | 0.32 0.03 | 0.31 0.04 | 0.29 0.03 |
| $\mu$ | 0.01 0.00 | 0.02 0.04 | 0.01 0.07 | 0.01 0.01 | 0.01 0.01 |

**Table 9** DIC results for indicated models. The three case studies.

| Model | Case study I | | | Case study II | | | Case study III | | |
|---|---|---|---|---|---|---|---|---|---|
| | Dbar | pD | DIC | Dbar | pD | DIC | Dbar | pD | DIC |
| Small sample size ($\boldsymbol{y}_1$) | | | | | | | | | |
| iCAR | 473 | 19 | 492 | 573 | 13 | 586 | | | |
| Scaled iCAR | 473 | 19 | 492 | 573 | 14 | 587 | | | |
| pCAR | 469 | 23 | 492 | 571 | 16 | 587 | | | |
| LCAR | 469 | 22 | 491 | 572 | 15 | 587 | | | |
| BYM | 469 | 24 | 493 | 568 | 20 | 588 | | | |
| Scaled BYM | 469 | 24 | 493 | 568 | 20 | 588 | | | |
| MBYM | 471 | 22 | 493 | 573 | 15 | 588 | | | |
| Scaled MBYM | 471 | 22 | 493 | 573 | 15 | 588 | | | |
| Modest sample size ($\boldsymbol{y}_2$) | | | | | | | | | |
| iCAR | 671 | 45 | 715 | 962 | 95 | 1057 | | | |
| Scaled iCAR | 671 | 45 | 715 | 962 | 95 | 1057 | | | |
| pCAR | 668 | 49 | 717 | 960 | 97 | 1057 | | | |
| LCAR | 668 | 47 | 715 | 960 | 96 | 1056 | | | |
| BYM | 669 | 47 | 716 | 958 | 97 | 1055 | | | |
| Scaled BYM | 669 | 47 | 716 | 959 | 97 | 1056 | | | |
| MBYM | 669 | 46 | 715 | 958 | 98 | 1056 | | | |
| Scaled MBYM | 669 | 46 | 715 | 958 | 97 | 1055 | | | |
| Large sample size ($\boldsymbol{y}$) | | | | | | | | | |
| iCAR | | | | | | | 923 | 91 | 1014 |
| Scaled iCAR | | | | | | | 923 | 91 | 1014 |
| pCAR | | | | | | | 916 | 85 | 1001 |
| LCAR | | | | | | | 916 | 85 | 1001 |
| BYM | | | | | | | 923 | 90 | 1013 |
| Scaled BYM | | | | | | | 922 | 90 | 1012 |
| MBYM | | | | | | | 916 | 85 | 1001 |
| Scaled MBYM | | | | | | | 916 | 85 | 1001 |

**Table 10** WAIC results for indicated models. The three case studies.

| Model | Case study I | | | Case study II | | | Case study III | | |
|---|---|---|---|---|---|---|---|---|---|
| | -2 lppd | $2p_{\text{WAIC 2}}$ | WAIC | -2 lppd | $2p_{\text{WAIC 2}}$ | WAIC | -2 lppd | $2p_{\text{WAIC 2}}$ | WAIC |
| Small sample size ($\boldsymbol{y}_1$) | | | | | | | | | |
| iCAR | 458 | 35 | 493 | 561 | 25 | 586 | | | |
| Scaled iCAR | 458 | 35 | 493 | 561 | 25 | 586 | | | |
| pCAR | 452 | 42 | 494 | 558 | 29 | 587 | | | |
| LCAR | 453 | 39 | 491 | 559 | 28 | 587 | | | |
| BYM | 452 | 42 | 494 | 553 | 34 | 587 | | | |
| Scaled BYM | 452 | 41 | 493 | 553 | 34 | 587 | | | |
| MBYM | 454 | 40 | 494 | 560 | 27 | 587 | | | |
| Scaled MBYM | 455 | 38 | 493 | 560 | 27 | 587 | | | |
| Modest sample size ($\boldsymbol{y}_2$) | | | | | | | | | |
| iCAR | 649 | 63 | 712 | 924 | 121 | 1045 | | | |
| Scaled iCAR | 649 | 62 | 711 | 924 | 121 | 1045 | | | |
| pCAR | 646 | 63 | 709 | 922 | 118 | 1041 | | | |
| LCAR | 647 | 62 | 709 | 922 | 118 | 1040 | | | |
| BYM | 647 | 62 | 709 | 921 | 116 | 1037 | | | |
| Scaled BYM | 648 | 63 | 710 | 922 | 118 | 1039 | | | |
| MBYM | 647 | 62 | 709 | 921 | 116 | 1038 | | | |
| Scaled MBYM | 648 | 63 | 710 | 921 | 116 | 1037 | | | |
| Large sample size ($\boldsymbol{y}$) | | | | | | | | | |
| iCAR | | | | | | | 892 | 107 | 999 |
| Scaled iCAR | | | | | | | 892 | 107 | 999 |
| pCAR | | | | | | | 889 | 87 | 976 |
| LCAR | | | | | | | 889 | 88 | 977 |
| BYM | | | | | | | 892 | 104 | 996 |
| Scaled BYM | | | | | | | 892 | 104 | 996 |
| MBYM | | | | | | | 889 | 88 | 977 |
| Scaled MBYM | | | | | | | 889 | 87 | 976 |

**Table 11** Posterior estimates, median and standard deviation (sd), of the model parameters without covariate (0 covar.) or with five covariates (5 covar.). Scaled BYM($\sigma_s, \sigma_h$) (BYM$^\dagger$) or scaled MBYM($c, \sigma$) (MBYM$^\dagger$) or pCAR is the risk model. For BYM$^\dagger$, $c = \sqrt{\sigma_s^2/(\sigma_s^2+\sigma_h^2)}$, $\sigma = \sqrt{\sigma_s^2+\sigma_h^2}$; for MBYM$^\dagger$, $\sigma_s = \sigma\sqrt{c}$, $\sigma_h = \sigma\sqrt{1-c}$. The five covariates are scores of: Private transportation to work ($\boldsymbol{x}_1$), Age 55-64 ($\boldsymbol{x}_2$), Education less than high school ($\boldsymbol{x}_3$), Colleage education ($\boldsymbol{x}_4$), and Unemployment ($\boldsymbol{x}_5$). The case study III.

| | BYM$^\dagger$ | BYM$^\dagger$ | MBYM$^\dagger$ | MBYM$^\dagger$ | pCAR | pCAR |
|---|---|---|---|---|---|---|
| Para. | 0 covar. | 5 covar. | 0 covar. | 5 covar. | 0 covar. | 5 covar. |
| | median sd | median sd | median sd | median sd | median sd | median sd |
| $\beta_0$ | 0.00 0.01 | 0.00 0.01 | 0.00 0.01 | 0.00 0.01 | 0.02 0.04 | 0.01 0.02 |
| $\beta_1$ | | 0.84 0.69 | | 0.87 0.69 | | 0.90 0.67 |
| $\beta_2$ | | -4.53 1.03 | | -4.59 1.05 | | -4.39 1.01 |
| $\beta_3$ | | 3.07 0.80 | | 3.18 0.81 | | 3.55 0.77 |
| $\beta_4$ | | 1.10 0.65 | | 1.03 0.68 | | 0.93 0.66 |
| $\beta_5$ | | -2.69 1.69 | | -2.90 1.65 | | -3.93 1.52 |
| $c$ | 0.93 0.13 | 0.63 0.28 | 0.81 0.15 | 0.50 0.20 | 0.79 0.12 | 0.47 0.23 |
| $\sigma$ | 0.22 0.02 | 0.17 0.02 | 0.20 0.02 | 0.16 0.02 | 0.36 0.03 | 0.31 0.03 |
| $\sigma_s$ | 0.21 0.03 | 0.13 0.05 | 0.18 0.03 | 0.11 0.04 | | |
| $\sigma_h$ | 0.06 0.04 | 0.10 0.04 | 0.09 0.03 | 0.11 0.03 | | |
| Deviance | 922 | 922 | 916 | 917 | 916 | 916 |
| pD | 90 | 89 | 85 | 84 | 85 | 85 |
| DIC | 1012 | 1011 | 1001 | 1001 | 1001 | 1001 |
| -2 lppd | 892 | 892 | 889 | 890 | 889 | 889 |
| $2p_{\text{WAIC 2}}$ | 104 | 103 | 87 | 88 | 87 | 89 |
| WAIC | 996 | 995 | 976 | 978 | 976 | 978 |

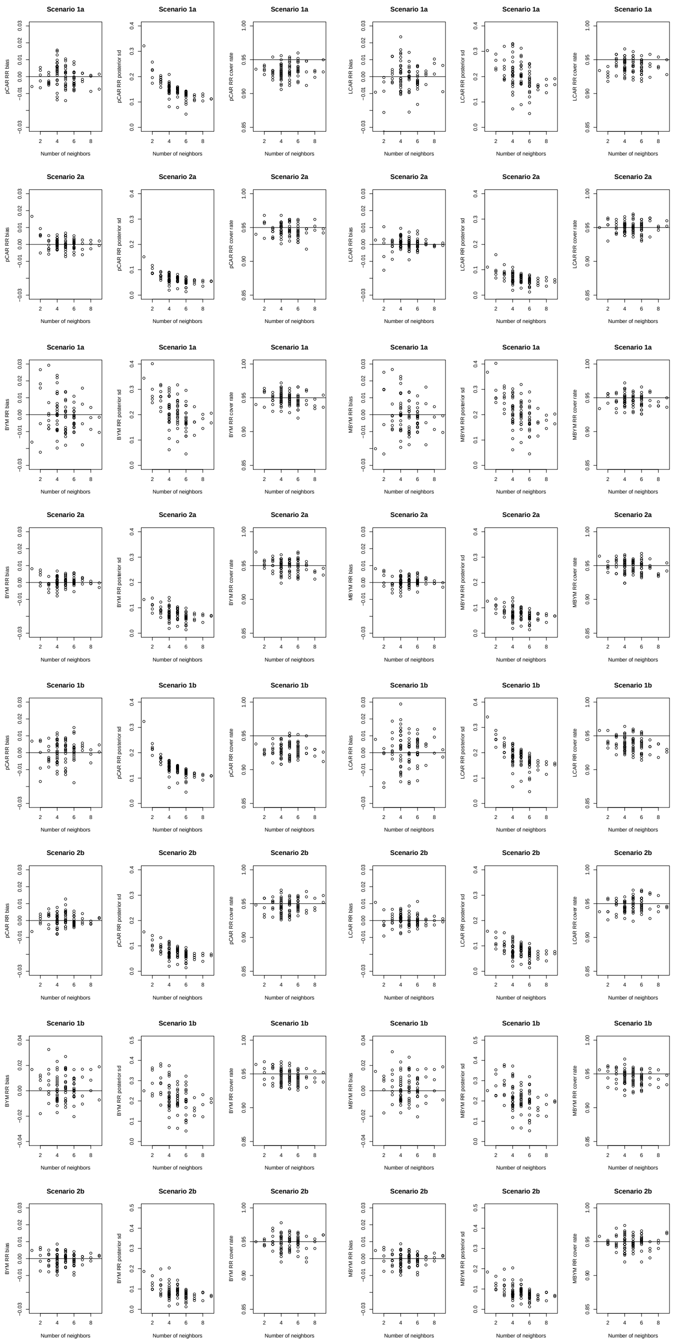

**Fig. 3** Posterior relative risk biases and coverage rates for the GLMM (1)-(4) with indicated risk model (pCAR, LCAR, BYM, MBYM), for simulation scenarios 1a, 2a, 1b, and 2b.

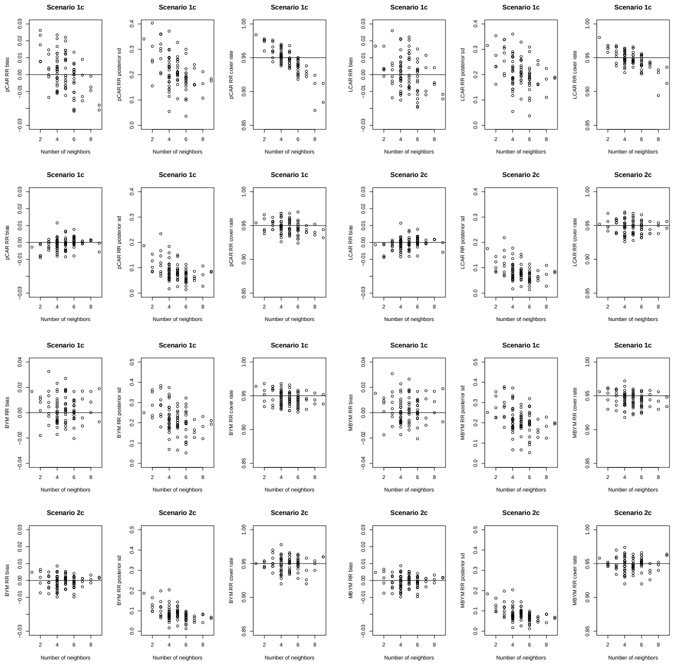


**Fig. 4** Posterior relative risk biases and coverage rates for the GLMM (1)-(4) with indicated risk model (pCAR, LCAR, BYM, MBYM), for the simulation scenarios 1c and 2c.

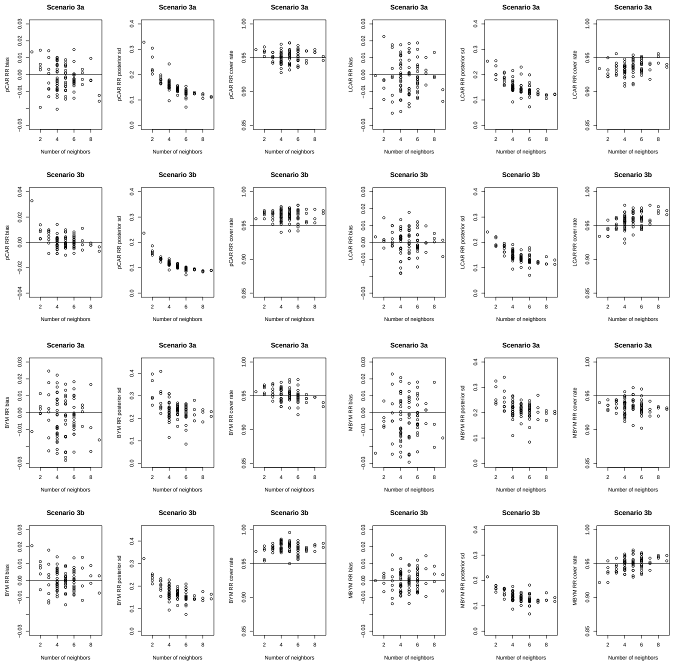

**Fig. 5** Posterior relative risk biases and coverage rates for the GLMM (1)-(4) with indicated risk model (pCAR, LCAR, BYM, MBYM), for the simulation scenarios 3a and 3b.

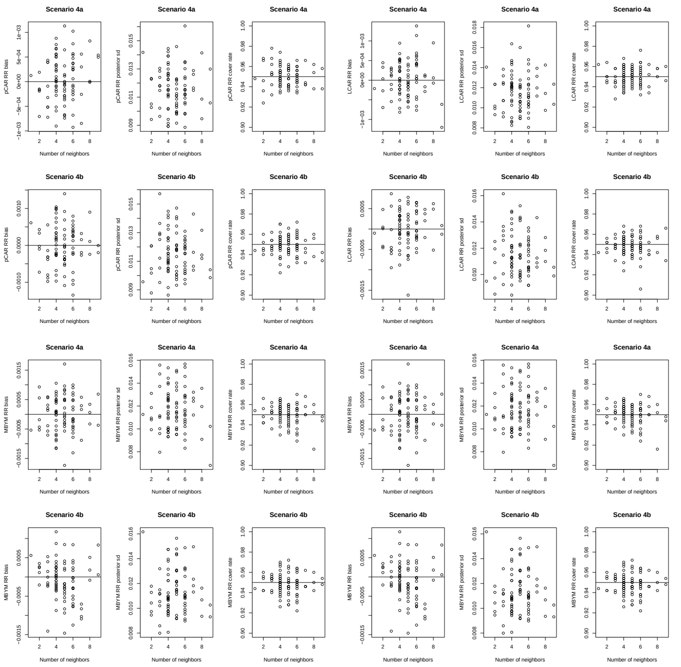


**Fig. 6** Posterior relative risk biases and coverage rates for the GLMM (1)-(4) with indicated risk model (pCAR, LCAR, MBYM), for the simulation scenarios 4a and 4b.

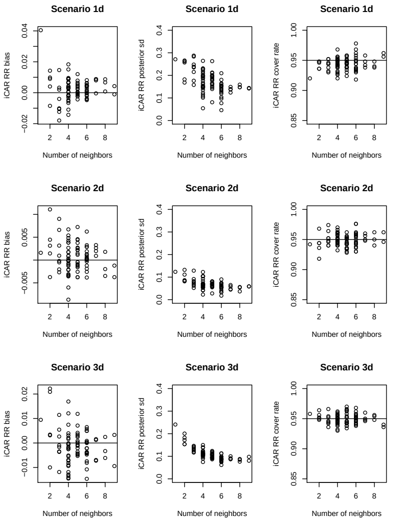


**Fig. 7** Posterior relative risk biases and coverage rates for the GLMM (1)-(4) with iCAR risk model, for the simulation scenarios 1d, 2d, and 3d, respectively.

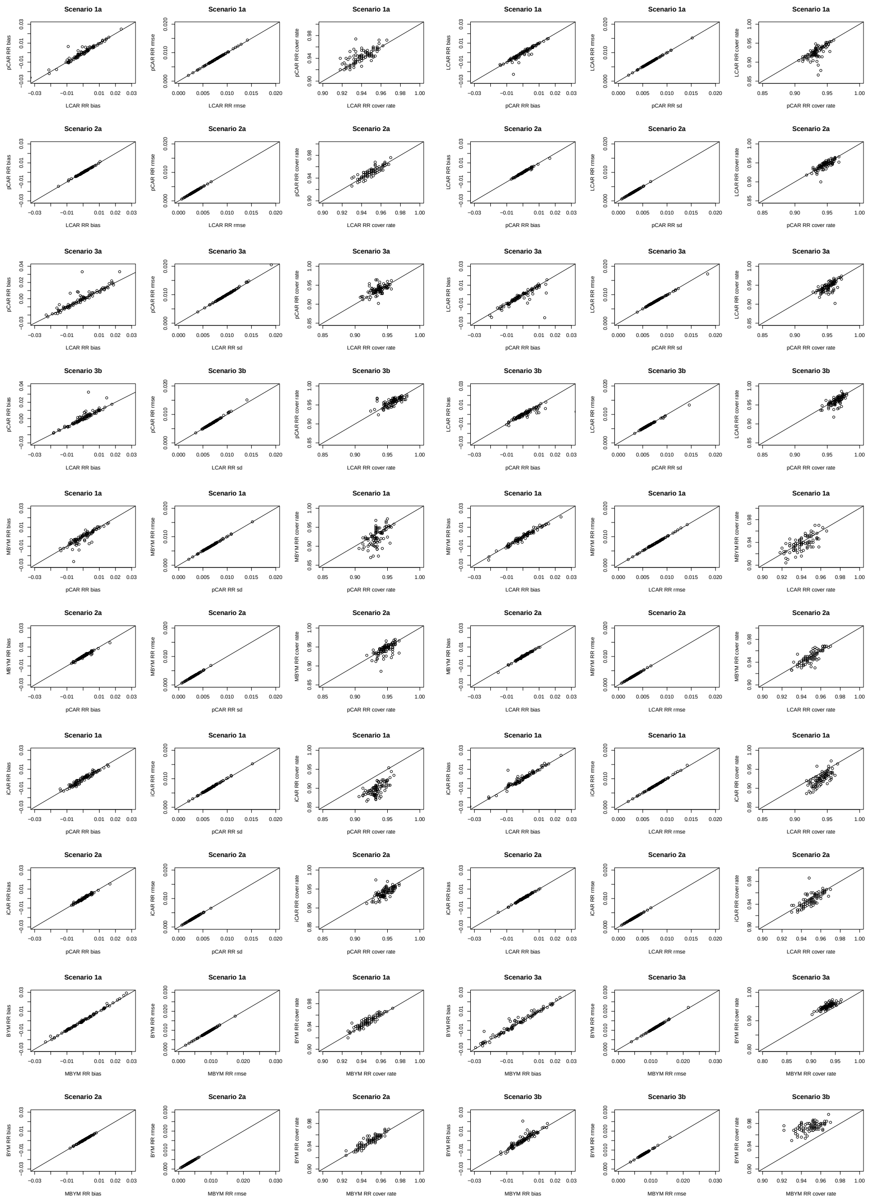

**Fig. 8** Simulation results of posterior relative risk prediction for indicated models: x-axis - results of the true risk model, y-axis: results of the fitted risk model, fitted to simulated data of the true risk model. rmse: square-root of mean square errors, cover rate: 95% credible interval coverage rate.

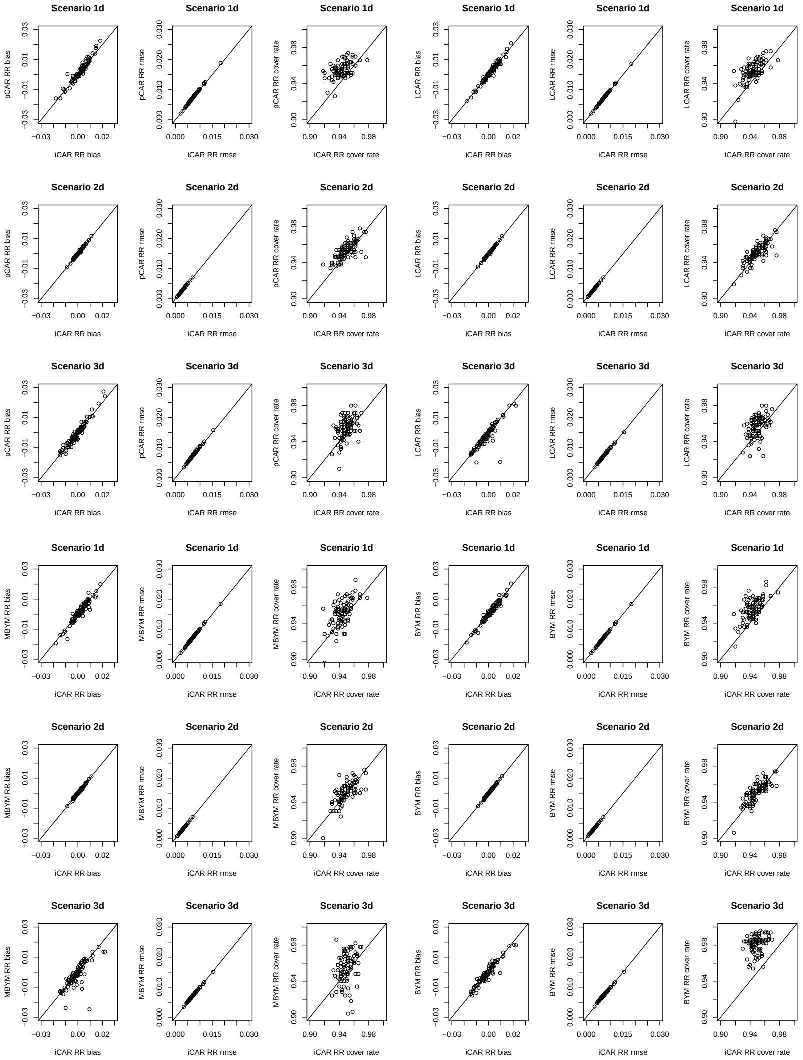


**Fig. 9** Simulation results of posterior relative risk prediction for indicated models: x-axis - results of the true risk model, y-axis: results of the fitted risk model, fitted to simulated data of the true model. rmse: square-root of mean square errors, cover rate: 95% credible interval coverage rate.

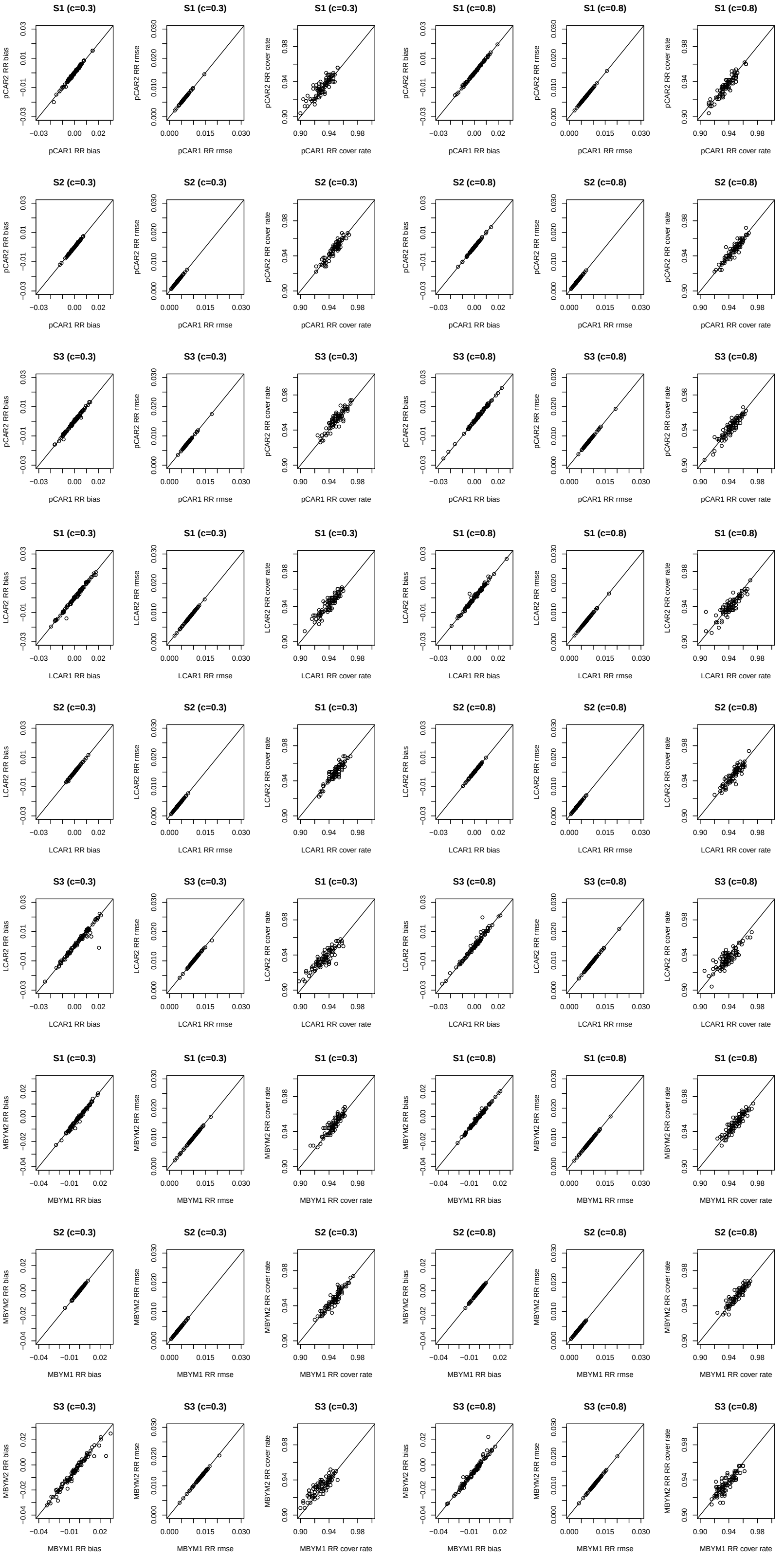


**Fig. 10** Simulation results of posterior relative risk prediction for the indicated simulation scenarios: S1 - Scenario 1, S2 - Scenario 2, S3 - Scenario 3. LCAR1 or MBYM1: model estimated under non-information prior $c \sim \text{Beta}(1, 1)$; LCAR2 or MBYM2: model estimated under information prior $c \sim \text{Beta}(3, 6)$ for true $c = 0.3$ or $c \sim \text{Beta}(8, 3)$ for true $c = 0.8$.

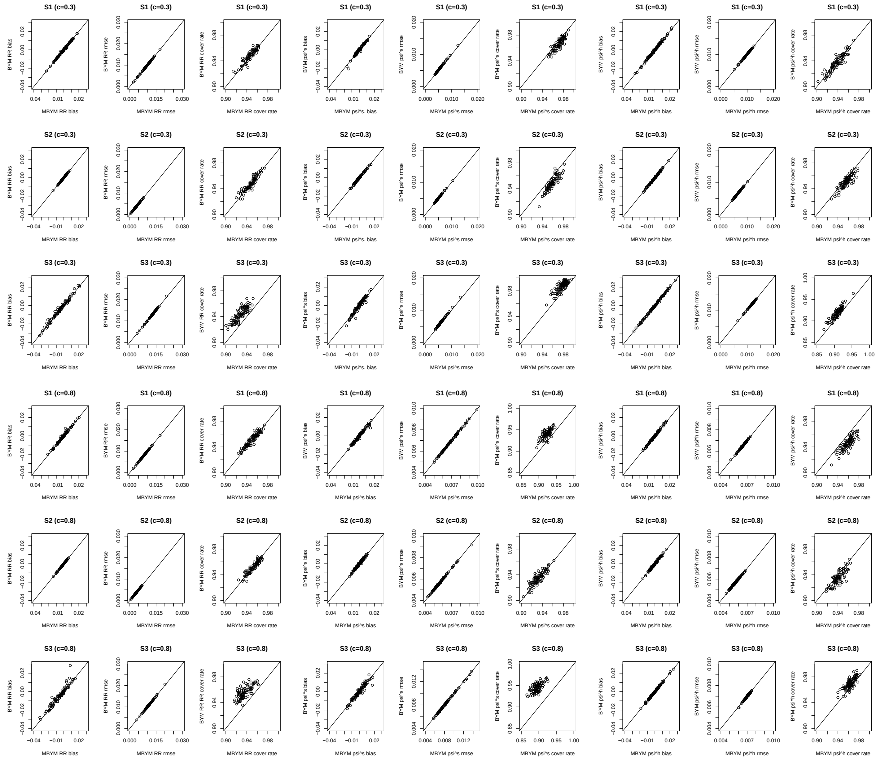

**Fig. 11** Posterior prediction of relative risks (RR) and components $\boldsymbol{\psi}^s$ and $\boldsymbol{\psi}^h$ under BYM versus MBYM. rmse: square-root of mean square errors, cover rate: 95% credible interval coverage rate.

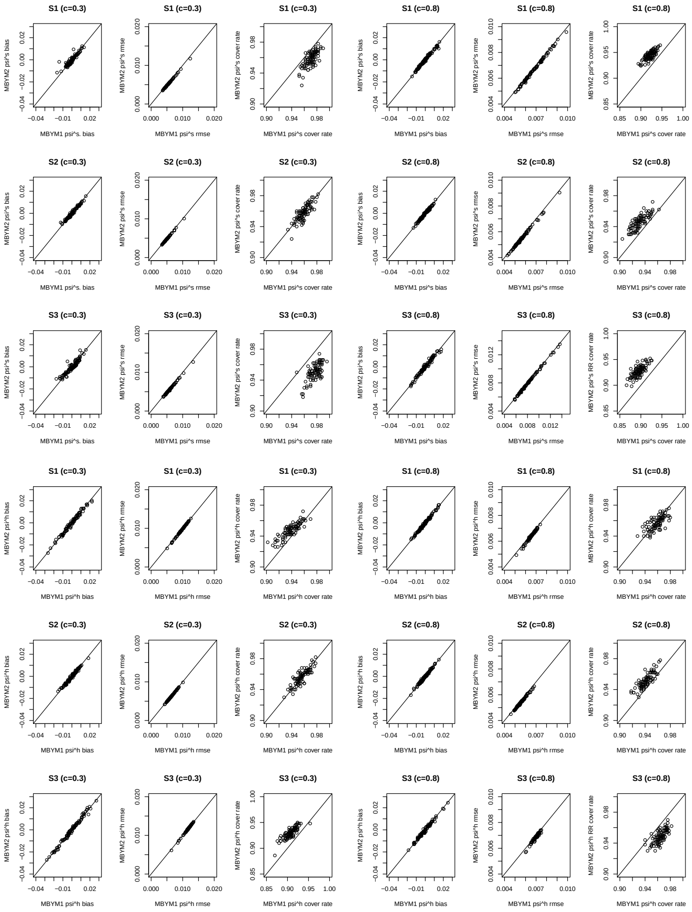

**Fig. 12** Posterior sensitivity of estimation and inference of spatial and non-spatial components $\boldsymbol{\psi}^s$ and $\boldsymbol{\psi}^h$ to spatial parameter prior specifications. MBYM1: model estimated under non-information prior $c \sim \text{Beta}(1,1)$; MBYM2: model estimated under information prior $c \sim \text{Beta}(3,6)$ for true $c = 0.3$ or $c \sim \text{Beta}(8,3)$ for true $c = 0.8$.

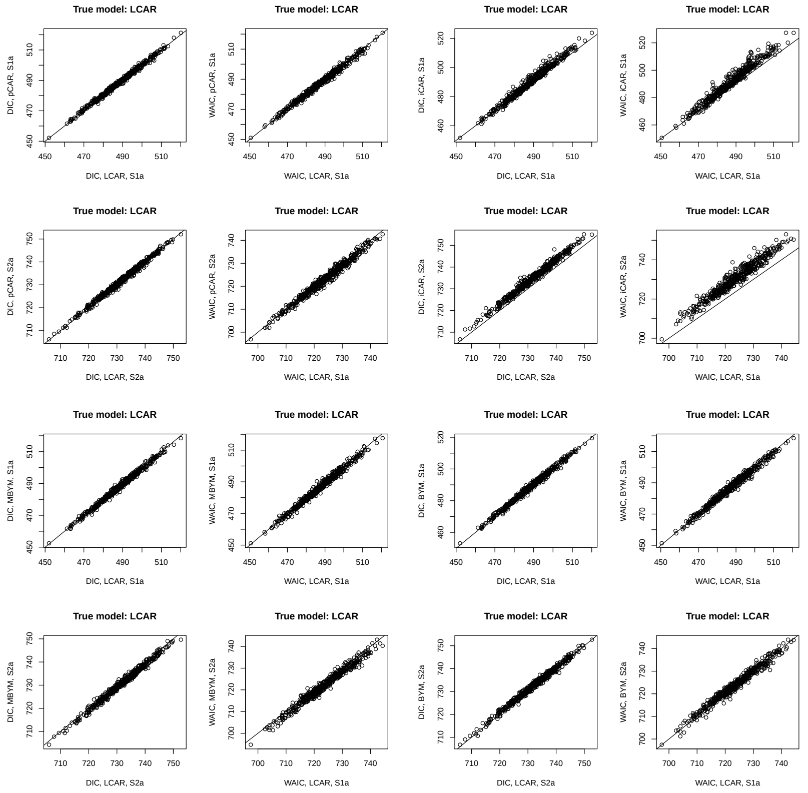


**Fig. 13** Simulation results of the deviance information criterion (DIC) and the widely applicable information criterion (WAIC) scores for indicated models: x-axis - results of the true risk model, y-axis: results of the fitted risk model, fitted to simulated data of the true risk model LCAR.

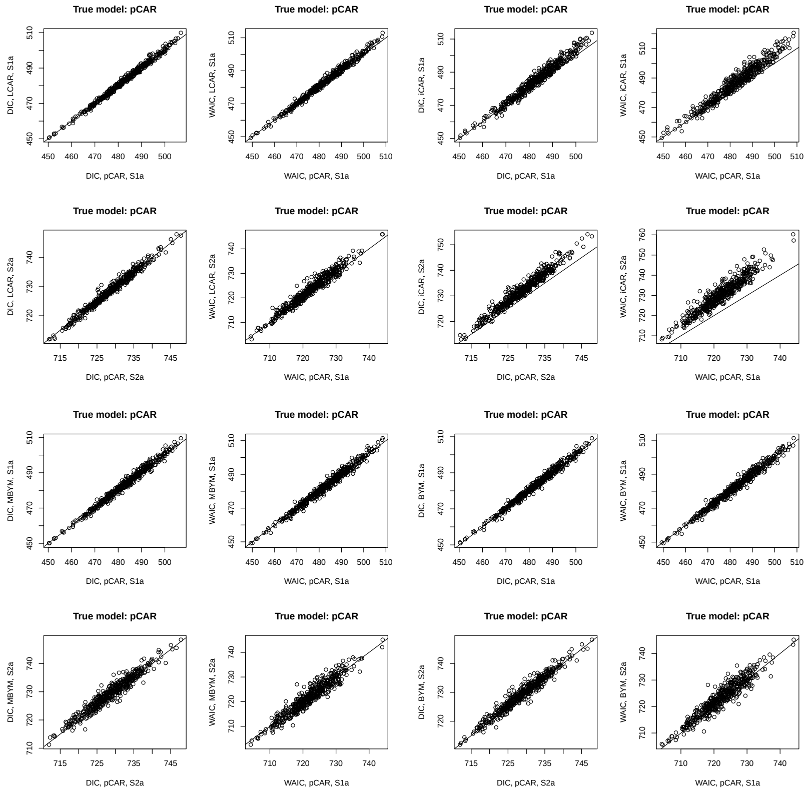


**Fig. 14** Simulation results of the deviance information criterion (DIC) and the widely applicable information criterion (WAIC) scores for indicated models: x-axis - results of the true risk model, y-axis: results of the fitted risk model, fitted to simulated data of the true risk model pCAR.

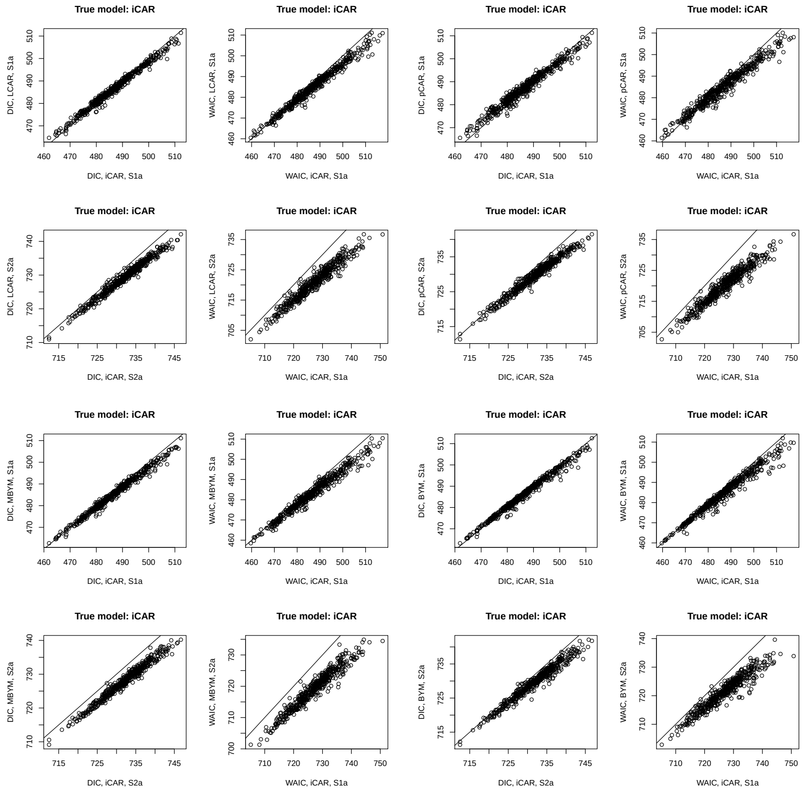


**Fig. 15** Simulation results of the deviance information criterion (DIC) and the widely applicable information criterion (WAIC) scores for indicated models: x-axis - results of the true risk model, y-axis: results of the fitted risk model, fitted to simulated data of the true risk model iCAR.

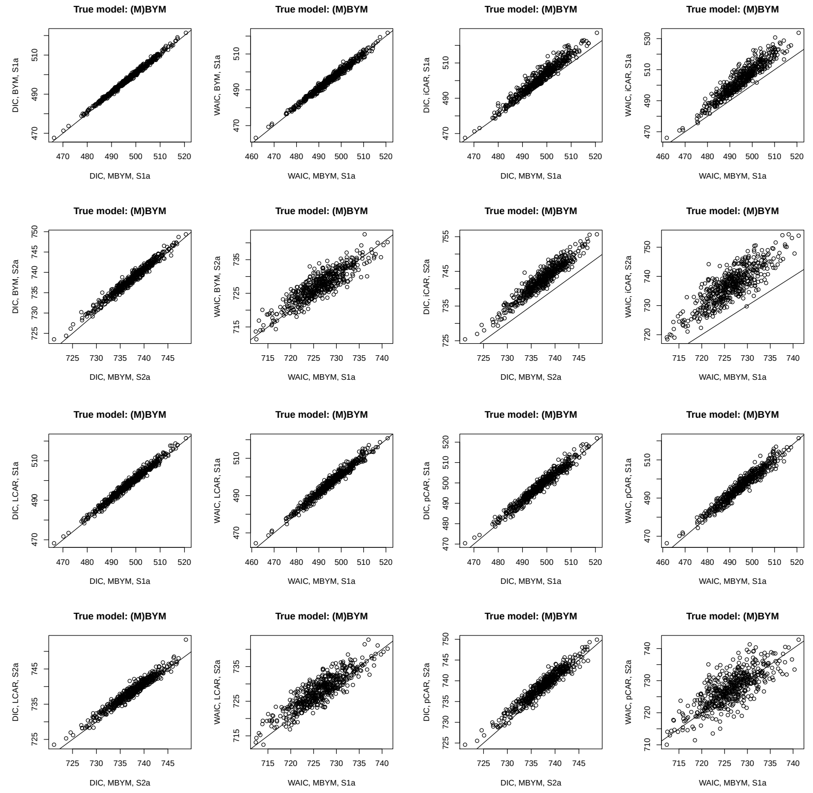


**Fig. 16** Simulation results of the deviance information criterion (DIC) and the widely applicable information criterion (WAIC) scores for indicated models: x-axis - results of the true risk model, y-axis: results of the fitted risk model, fitted to simulated data of the true risk model (M)BYM.

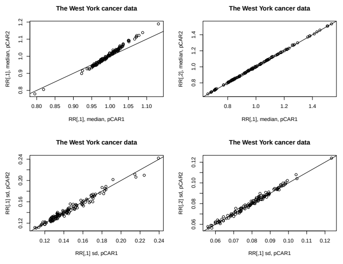


**Fig. 17** Posterior risk predictions (median and standard deviation) under informative or non-informative hyper-prior for $c \in (0, 1)$. pCAR1: model estimated under non-information prior $c \sim \text{Beta}(0, 1)$; pCAR2: model estimated under information prior $c \sim \text{Beta}(8, 3)$.

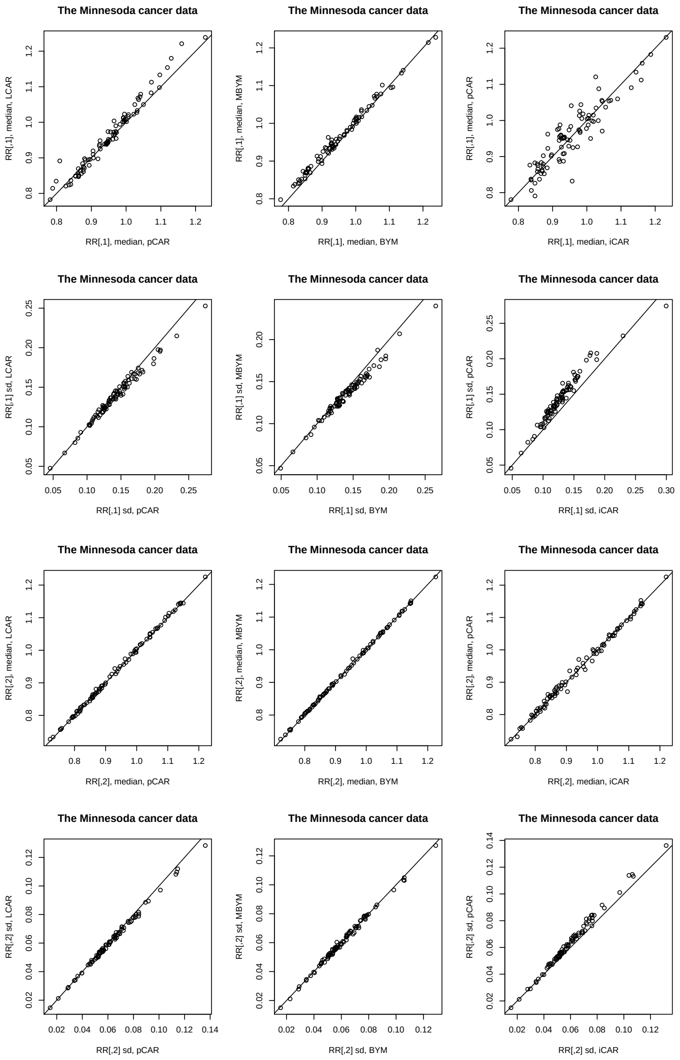


**Fig. 18** Comparisons among the five CAR models in terms of areal-level posterior relative risk predictions: median - posterior median, sd - posterior standard deviation. Results of the Case Study I.

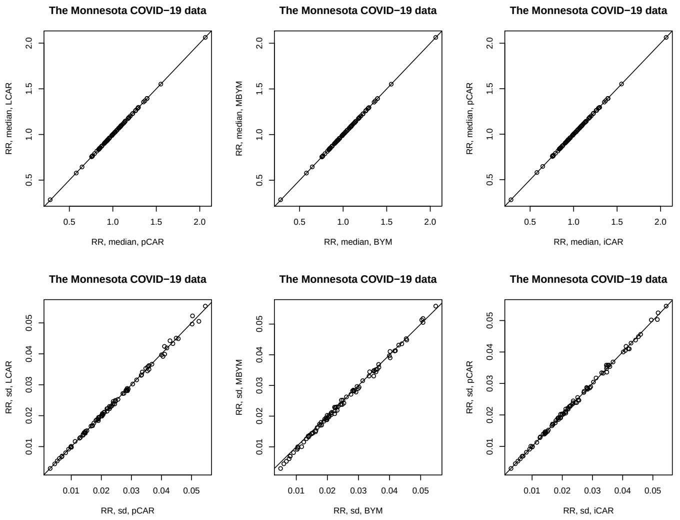


**Fig. 19** Comparisons among the five CAR models in terms of areal-level posterior relative risk predictions: median - posterior median, sd - posterior standard deviation. Results of the Case Study III.

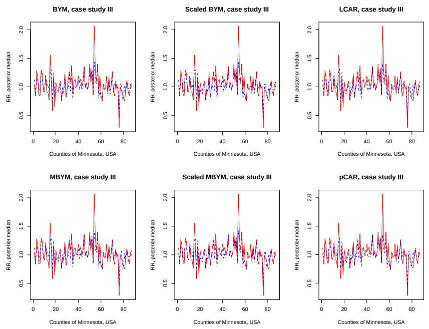


**Fig. 20** Posterior estimates, posterior median, of relative risks in the indicated spatial GLMM (1)-(4) without or with covariates. Solid (red) line: without covariates, dashed (blue) line: with covariates. The case study III
